# Integrative Analysis of the Metabolome and Transcriptome Provides Insights into the Mechanisms of Flavonoid Biosynthesis in Quinoa Seeds at Different Developmental Stages

**DOI:** 10.3390/metabo12100887

**Published:** 2022-09-22

**Authors:** Qianchao Wang, Lan Yao, Qunying Li, Heng Xie, Yirui Guo, Tingzhi Huang, Xuesong Zhang, Junna Liu, Ping Zhang, Li Li, Peng Qin

**Affiliations:** 1 College of Agronomy and Biotechnology, Yunnan Agricultural University, Kunming 650201, China; 2 College of Foresty and Horticulture, Hubei Minzu University, Enshi 445000, China; 3 Dali Seed Management Station, Dali 671000, China

**Keywords:** quinoa, grain development, flavonoid compound, metabolome, transcriptome

## Abstract

Quinoa (*Chenopodium quinoa* Willd.) is a crop with high nutritional and health benefits. Quinoa seeds are rich in flavonoid compounds; however, the mechanisms behind quinoa flavonoid biosynthesis remain unclear. We independently selected the high-generation quinoa strain ‘Dianli-3260′, and used its seeds at the filling, milk ripening, wax ripening, and mature stages for extensive targeted metabolome analysis combined with joint transcriptome analysis. The results showed that the molecular mechanism of flavonoid biosynthesis in quinoa seeds was mainly concentrated in two pathways: “flavonoid biosynthesis pathway” and “flavone and flavonol biosynthesis pathway”. Totally, 154 flavonoid-related metabolites, mainly flavones and flavonols, were detected in the four development stages. Moreover, 39,738 genes were annotated with KEGG functions, and most structural genes of flavonoid biosynthesis were differentially expressed during grain development. We analyzed the differential flavonoid metabolites and transcriptome changes between the four development stages of quinoa seeds and found that 11 differential flavonoid metabolites and 22 differential genes were the key factors for the difference in flavonoid biosynthesis. This study provides important information on the mechanisms underlying quinoa flavonoid biosynthesis, the screening of potential quinoa flavonoid biosynthesis regulation target genes, and the development of quinoa products.

## 1. Introduction

Quinoa (*Chenopodium quinoa* Willd.), also known as Indian wheat, grey rice, and golden millet, is an annual self-pollinated dicotyledonous herbaceous crop in the subfamily Chenopodiaceae of Amaranthaceae [1,2]. It is native to the alpine mountains of the Andes in South America, growing at an altitude of 2800–5000 m. It has been consumed and cultivated for more than 5000–7000 years, and demonstrates tolerance to cold, salinity, drought, and infertile soil, as well as other quality characteristics [3]. Quinoa seeds are rich in flavonoids, proteins, amino acids, minerals, polyphenols, vitamins, dietary fiber, unsaturated fatty acids, and other components [4,5,6], The ancient Incas called quinoa the “mother of grains,” and they used its seeds as a traditional food because of its unique nutritional qualities and functional food properties; [3,7,8]. The Food and Agriculture Organization of the United Nations considers quinoa as a complete nutritional food source for human beings, officially recommending it as the most suitable “fully nutritious food” for human consumption [9,10]. Quinoa has also been listed as the ideal “space food” for human migration to outer space in the future [9,11].

Plants abound in flavonoid compounds. Their synthetic pathways are relatively conserved and are among the most well-studied biosynthetic pathways for plant secondary metabolites [12]. The synthesis of flavonoids originates from the phenylpropane metabolic pathway, and the production of the intermediate of this pathway, phenylalanine, is catalyzed by phenylalanine lyase (PAL), cinnamic acid-4-hydroxylase (C4H), and 4-coumaroyl-coenzyme A ligase (4CL) to produce 4-coumaroyl-CoA, followed by chalcone synthase (CHS), chalcone isomerase (CHI), flavanone 3-hydroxylase (F3H), and flavonol synthase (FLS), and then generates various flavonoids under the action of CHS, CHI, F3H, FLS, and anthocyanidin synthase (ANS) [13]. Moreover, various transcription factors (TFs), such as myeloblastosis (MYB), basic helix–loop–helix (bHLH), and WD40 proteins, have important regulatory effects on flavonoid synthesis [14]. The biosynthetic pathways of flavonoids are well studied in many plant species [15,16,17,18,19]. Flavonoids have antioxidant properties and have the potential to counter some diseases [20,21]. They play an important role in the growth and development of plants [22,23] and also act as natural barriers for plants to cope with various biological and abiotic stresses [24,25]. Quinoa is being rediscovered as a new crop, and its seeds are increasingly becoming a major part of people’s consumption. Quinoa seeds are also rich in antioxidants, which makes quinoa a great candidate for functional food development [10,11,12]. Thus far, there have been few physiological studies on quinoa seed flavonoids [26,27]. In addition to our previous reports on flavonoid biosynthesis in the seeds of different varieties at maturity, there has been no other report on the biosynthesis mechanism of quinoa flavonoids, especially the combination of metabolomics and transcriptomics. Previously, we only studied seeds at the mature stage. Therefore, to take the approach forward, we comprehensively analyzed the changes in flavonoid metabolites in four development stages of quinoa seeds (filling, milking, dough, and mature stages) using ultra high-performance liquid chromatography and tandem mass spectrometry (UPLC-MS/MS) in this study. Further, genes related to different development stages of quinoa seeds were screened by transcriptomics, and the molecular mechanisms of flavonoid biosynthesis in different development stages of quinoa seeds were clarified. The results of this study help deepen the understanding of the antioxidant components of quinoa and provide a valuable reference for the future development of quinoa products and selective breeding.

## 2. Results

### 2.1. Qualitative and Quantitative Analyses of Related Metabolites in Quinoa Seeds at Different Development Stages

**Figure 1:**
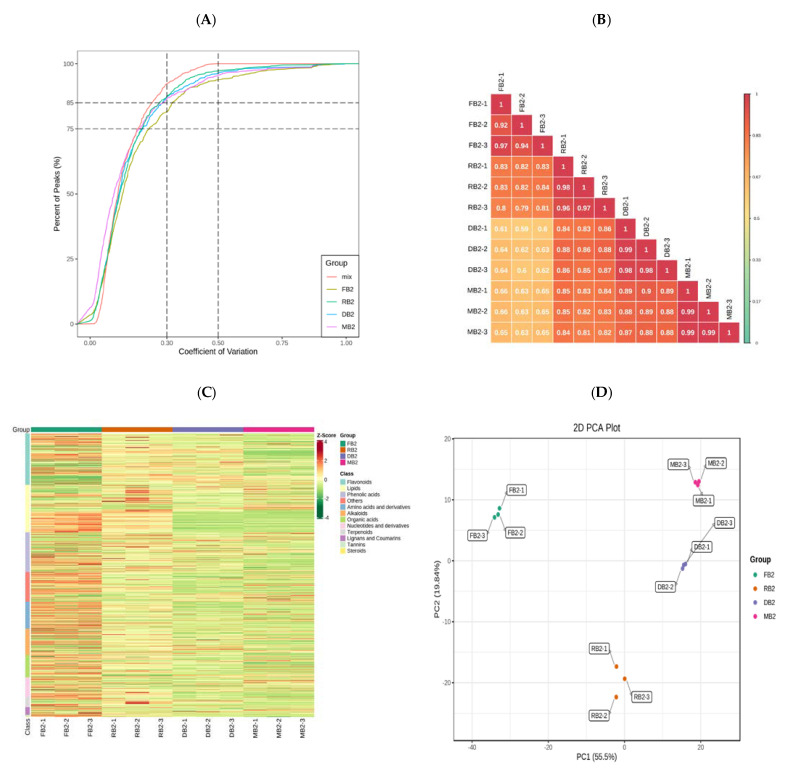
(**A**) Coefficient of variation (CV) value distribution of all samples. Note: the abscissa represents the CV value, the ordinate represents the proportion of the number of substances less than the corresponding CV value, and different colors represent different grouped samples. Mix represents QC samples, in which the CV values corresponding to the two reference lines perpendicular to the X−axis are 0.3 and 0.5, and the number of substances corresponding to the two reference lines parallel to the X−axis accounts for 75% and 85% of the total number of substances. The higher the proportion of substances with lower CV values of QC samples, the more stable are the experimental data. The proportion of substances with a CV value less than 0.5 in the QC samples is higher than 85%, indicating that the experimental data are relatively stable. The proportion of substances with a CV value less than 0.3 in the QC samples is higher than 75%, indicating that the experimental data are very stable.; (**B**) Correlation diagram between samples; (**C**) Overall cluster analysis heat map of sample; (**D**) PCA score diagram of mass spectrum data of each group of samples and quality control samples. Note: PC1 represents the first principal component, PC2 represents the second principal component, PC3 represents the third principal component, and percentages represents the interpretation rate of the principal component to the data set; each point in the figure represents a sample, and the samples of the same group are represented by the same color.

Twelve samples were selected for this project and divided into four groups for metabolic studies, with each group comprising three biological replicates. Metabolite quantification was accomplished using triple quadrupole mass spectrometry in multiple reaction monitoring (MRM, Figure S1) mode for accurate and reproducible quantification. The overlap of the total ion flow plots (TIC plots) analyzed by mass spectrometric detection of the different QC samples was demonstrated, and the results showed a high overlap of the curves of the total ion flow for metabolite detection, indicating good instrument stability and technical reproducibility, providing an important guarantee for the true reliability of the data (Figure S2). The distribution of the coefficient of variation (CV) values for all samples showed that the experimental data from this study were very stable (Figure 1A). Combined with the correlation map between samples (Figure 1B), the overall cluster analysis heat map of the samples (Figure 1C), and the PCA score plot (Figure 1D), the results showed that the biological repeatability within the sample group was good, and there were significant differences in flavonoid-related metabolites in quinoa seeds at different development stages. Qualitative and quantitative analysis of quinoa seed-related metabolites based on the KEGG Compound Database, MWDB, and multiple-reaction monitoring (MRM) was performed for a total of 154 flavonoid metabolites, including 3 chalcones, 16 flavanones, 5 flavanonols, 36 flavones, 73 flavonols, 12 flavonoid carbonosides, and 9 flavanols, and the RT was displayed (Table S1).

### 2.2. Analysis of Differences in Flavonoid-Related Metabolites in Quinoa Seeds at Different Developmental Stages

**Figure 2:**
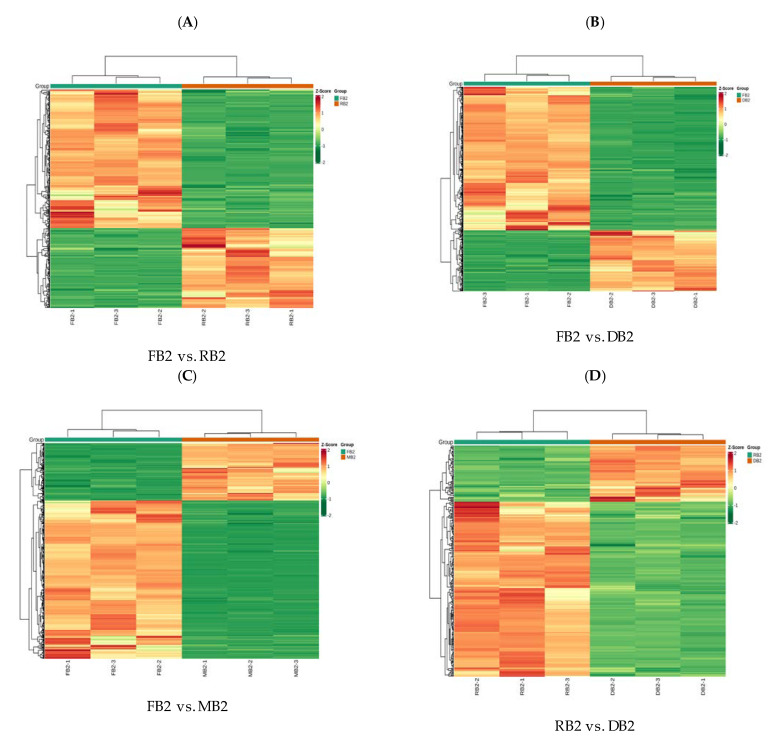

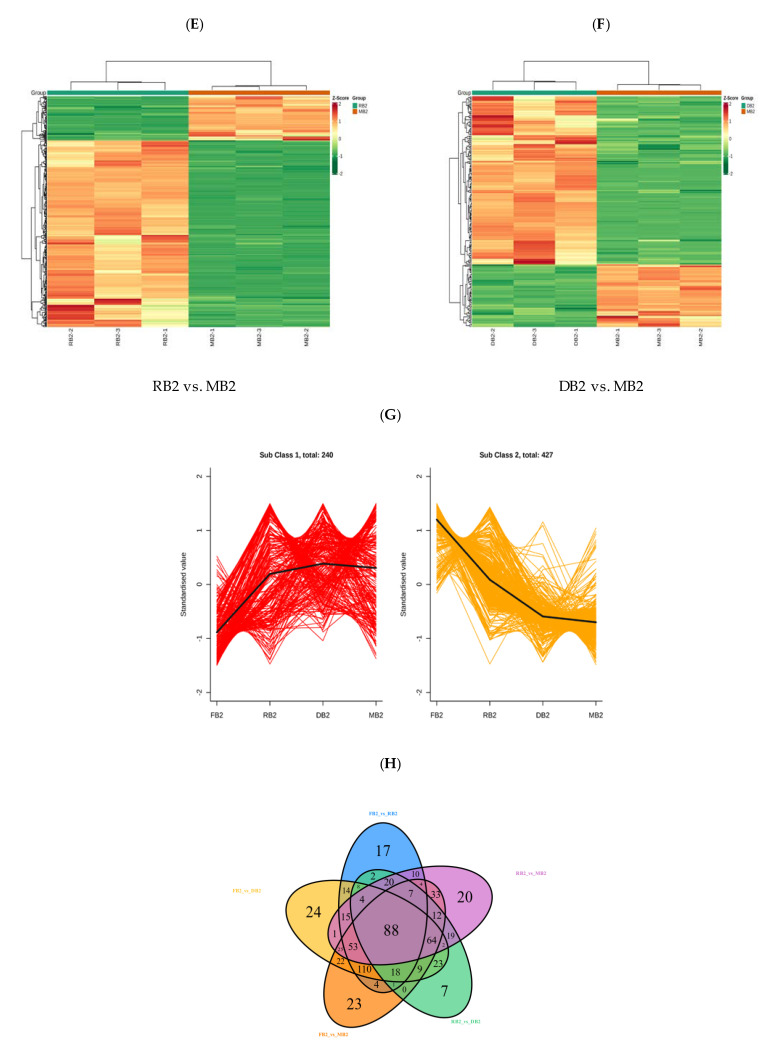
Differential accumulation of flavonoid metabolites during seed development in Quinoa nigra. (**A**–**F**) Heat maps of differential accumulation of flavonoid metabolites in FB2 vs. RB2, FB2 vs. DB2, FB2 vs. MB2, DB2 vs. MB2, RB2 vs. MB2, and RB2 vs. DB2, respectively; (**G**) K−means plot of relative content of differential metabolites; (**H**) Venn diagram of differential accumulation of flavonoid metabolites in different comparison groups; each circle in the figure represents a comparison group. Where the circles overlap, the numbers represent the number of common differential metabolites between the comparison groups. The numbers in the non−overlapping parts of the circles represent the number of unique differential metabolites of the comparison group.

Before performing difference analysis of relevant metabolites, we carried out principal component analysis (PCA) within each group and found that the intra-group variance of the samples was small (Figure S3). From the OPLS-DA, the variables with less correlation could be compared and the results showed that the Q2 values of all sub-groups were greater than 0.9 (*p* < 0.05), indicating that the constructed model was both reliable and realistic, with good predictive power (Figure S4). Based on the results of OPLS-DA, we analyzed the variable importance in projection (VIP) of the OPLS-DA model from the obtained multivariable, and selected the differentially accumulated metabolites of different groups. A total of 154 common flavonoid metabolites were detected in the four development stages of quinoa seeds. Most of these flavonoids were most abundant at the FB2, and least abundant at the MB2; for example, naringenin, phloretin, dihydroquercetin, quercetin, gallocatechin, and epigallocatechin. P-coumaroylshikimic acid and luteolo-side content were the highest in RB2; 3-O-[beta-D-xylosyl-(1->2)-beta-D-glucoside] and quercetin 3-(2G-xylosylrutinoside) content in the DB2 was higher than in the other stages, and the content of quercetin 3-(2G xylosylrutinoside) was also higher in the MB2, after that in the DB2. There were 86 different metabolites of flavonoids in FB2 vs. RB2, of which 41 (2 chalcones, 4 flavanones, 3 flavanonols, 6 flavones, 19 flavonols, 3 flavonoid carbonosides, and 4 flavanols) were down-regulated and 45 (1 chalcone, 3 flavanones, 1 flavanonol, 10 flavones, 25 flavonols, 4 flavonoid carbonosides, and 1 flavanol) were up-regulated (Figure 2A, Table S2); There were 98 different metabolites of flavonoids in FB2 vs. DB2, of which 52 (2 chalcones, 5 flavanones, 3 flavanonols, 9 flavones, 25 flavonols, 3 flavonoid carbonosides, and 5 flavanols) were down-regulated and 46 (1 chalcone, 5 flavanones, 1 flavanonol, 10 flavones, 19 flavonols, 4 flavonoid carbonosides, and 1 flavanol) were up-regulated (Figure 2B, Table S3). There were 97 different metabolites of flavonoids in FB2 vs. MB2, of which 61 (2 chalcones, 6 flavanones, 4 flavanonols, 11 flavones, 31 flavonols, 3 flavonoid carbonosides, and 5 flavanols) were down-regulated and 36 (1 chalcone, 3 flavanones, 8 flavones, 19 flavonols, 4 flavonoid carbonosides, and 1 flavanol) were up-regulated (Figure 2C, Table S4); There were 20 different metabolites of flavonoids in RB2 vs. DB2, of which 17 (4 flavanones, 1 flavanonol, 3 flavones, 7 flavonols, 1 flavonoid carbonoside, and 1 flavanol) were down-regulated and 3 (2 flavonols and 1 flavone) were up-regulated (Figure 2D, Table S5). There were 64 different metabolites of flavonoids in RB2 vs. MB2, of which 61 (1 chalcone, 5 flavanones, 4 flavanonols, 9 flavones, 34 flavonols, 3 flavonoid carbonosides, and 5 flavanols) were down-regulated and 3 (2 flavones and 1 flavonol) were up-regulated (Figure 2E, Table S6). There were 64 different metabolites of flavonoids in DB2 vs. MB2, of which 45 (3 flavanones, 4 flavanonols, 6 flavones, 26 flavonols, 2 flavonoid carbonosides, and 4 flavanols) were down-regulated and two (2 flavones) were up-regulated (Figure 2F, Table S7).

To study the change trends of the relative metabolite contents in different samples, the relative content of different metabolites was standardized and centralized, and then K-means clustering analysis was performed. It was found that most flavonoids were mainly concentrated in the sub-class 1 and sub-class 2 clusters, and the flavonoids in sub-class 1 showed a gradual growth trend with the development period, whereas those in sub-class 2 gradually decreased with the development period (Figure 2G, Table S8); Venn diagrams identified a total of 88 differential metabolites in all subgroups: FB2 vs. RB2, FB2 vs. DB2, FB2 vs. MB2, RB2 vs. DB2, DB2 vs. MB2 had 17, 24, 23, 7, and 20 differential metabolites, respectively, of which the flavonoid metabolites were dominated by flavones and flavonols (Figure 2H, Table S9). Differential flavonoid metabolites identified in the different comparison groups were further annotated using the KEGG database. The results showed that the differential flavonoid metabolites between the different comparison groups were mainly involved in the flavonoid biosynthesis and flavone and flavonol biosynthesis pathways.

### 2.3. Transcriptome Analysis of Quinoa Seeds at Different Developmental Stages

After raw data filtering, sequencing error rate checking and guanine-cytosine content distribution checking, a total of 82.62 Gb of clean data was obtained, and the clean data of each sample reached 6 Gb, with the percentage of Q30 bases at 92% and above (Table S10). The comparison efficiency is the most direct embodiment of the utilization of transcriptome data. The proportion of sequenced reads successfully matched to the genome was higher than 84%, and the matching efficiency was higher than 80%, indicating that the sequencing results were accurate and ready for further analysis (Table S11).

**Figure 3:**
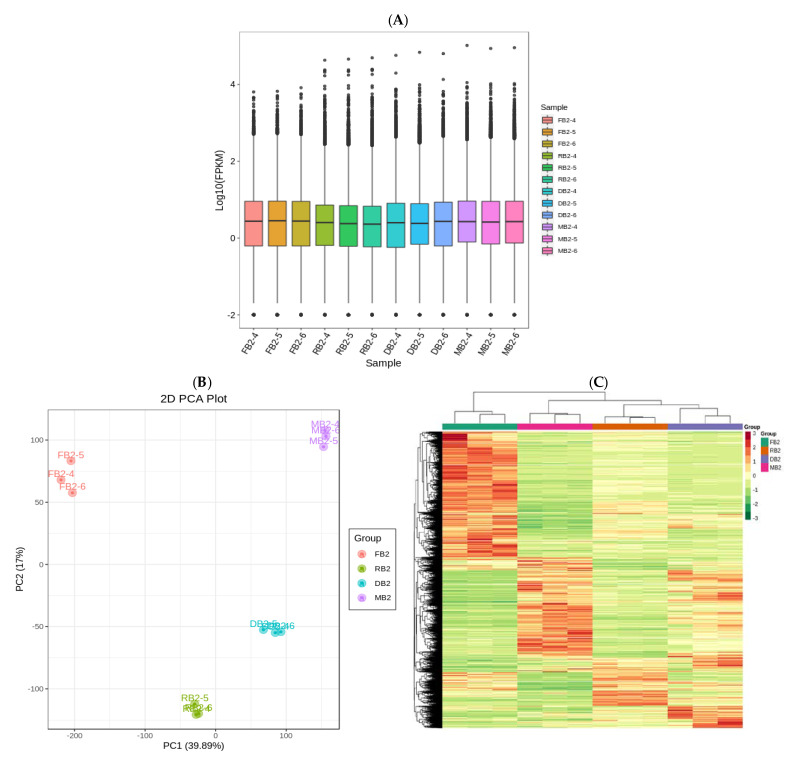
(**A**) Expression box line diagram. The abscissa in the figure represents different samples; the ordinate represents the logarithm of the sample FPKM expression. The figure measures the expression level of each sample from the perspective of overall dispersion of expression quantity. (**B**) Principal component analysis diagram. Percentages represent the interpretation rate of the principal component to the data set; each point in the figure represents a sample, and samples of the same group are represented by the same color. (**C**) Differential gene clustering heat map. The abscissa represents the sample name and hierarchical clustering results, and the ordinate represents the differential genes and hierarchical clustering results. Red indicates high expression, and green indicates low expression.

Generally, the FPKM values of the protein coding gene expression levels that were sequenced ranged from 10^−2^–10^−4^ to six orders of magnitude. From the box diagram, we can see that the dispersion of gene expression level distribution of each sample in this study was small, and the overall gene expression level was high. The FPKM distribution box diagram of the 12 samples is shown in Figure 3A, showing the concentration of gene abundance in the quinoa seeds at different developmental stages as the expression level changes. The principal component analysis (PCA) plot (Figure 3B) indicated that the flavonoid biosynthesis genes did not differ much within groups and were relatively concentrated and revealed a clear separation among developing quinoa seed samples at different stages, and the 56.89% variance among the samples could be explained by PCA1 (39.89%) and PCA2 (17%), indicating that flavonoid biosynthesis genes present a dynamic change pattern during quinoa seeds at different developmental stages. The screening conditions for differential genes were |log2fold change| ≥ 1 and FDR < 0.05, and after extracting the centralized and normalized FPKM expressions of the differential genes, performing hierarchical cluster analysis, plotting the cluster heat map of each differential grouping, and combining with the PCA, it could be seen there were obvious differences in the expression of all genes expression in quinoa seeds at different developmental periods; with the advancement of development period, the number of up-regulated genes gradually decreased and the number of down regulated genes gradually increased (Figure 3C). For samples with biological replicates, DESeq2 is suitable for performing differential expression analysis between sample groups to obtain the set of DEGs between two biological conditions. After the analysis of DEGs has been completed using DESeq2, the total number of DEGs, the number of up-regulated genes and the number of down-regulated genes in each group were counted (Table 1).

### 2.4. Functional Annotation and Enrichment Analysis of Differentially Expressed Genes

The genes detected in this experiment were annotated in the KEGG, GO, NR, Swiss-Prot, KOG, Pfam, TrEMBL, and TF databases. The results showed that the function of KEGG was annotated to 39,738 genes, GO was annotated to 38,192 genes, NR was annotated to 53,873 genes, Swiss-Prot was annotated to 34,643 genes, KOG was annotated to 49,302 genes, Pfam was annotated to 45,315 genes, TrEMBL was annotated to 52,950 genes, and TF was annotated to 3409 genes.

**Figure 4:**
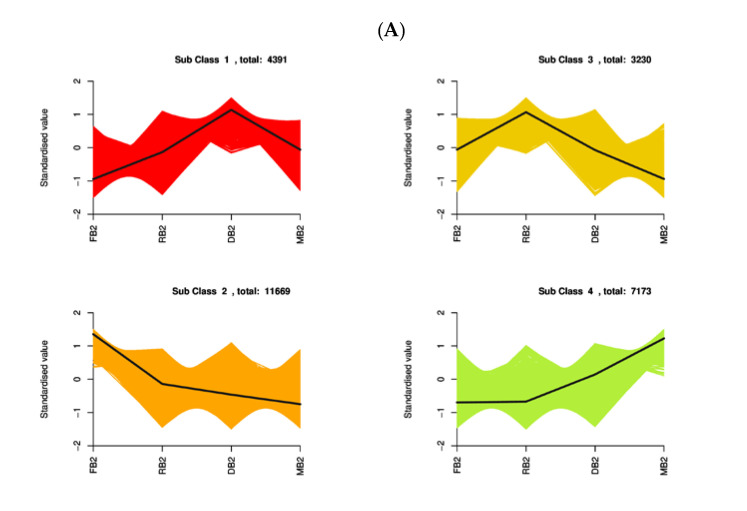

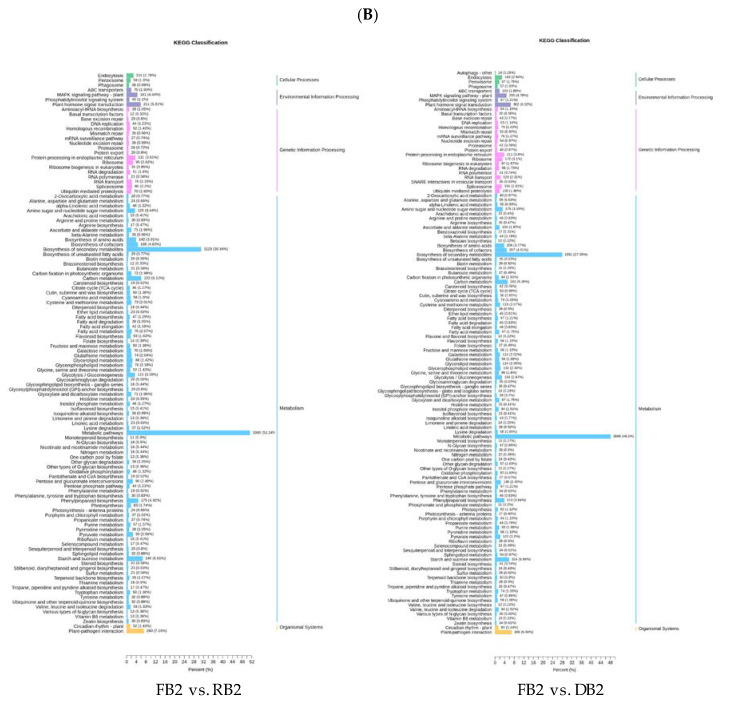

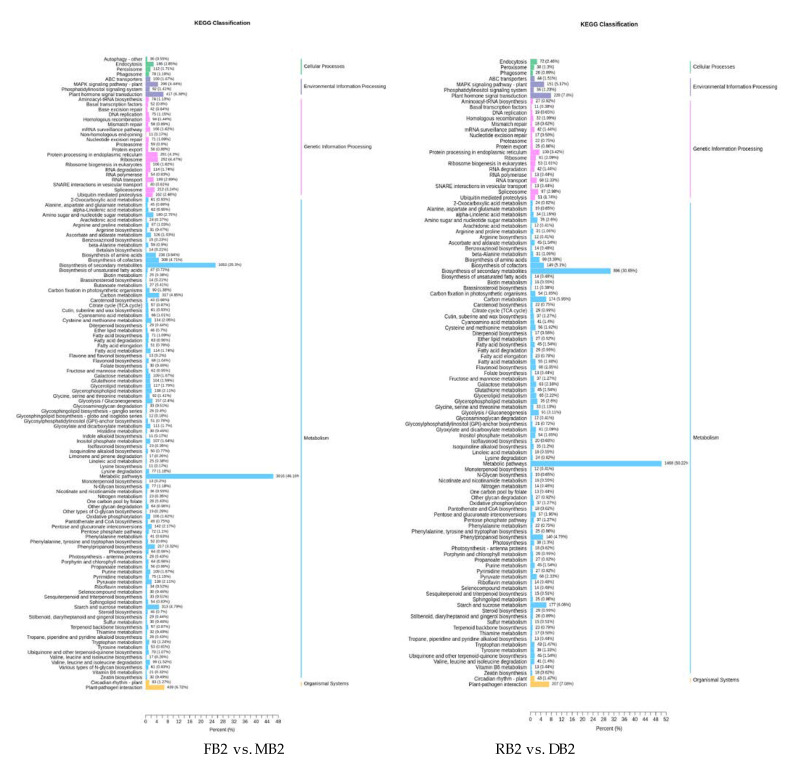

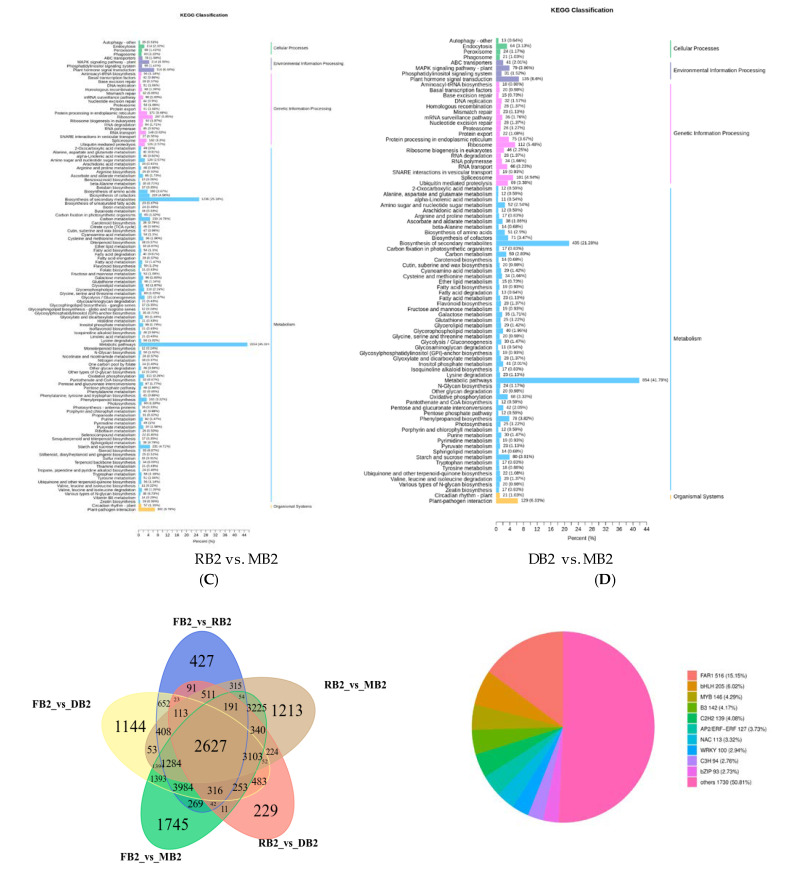
(**A**) K−means clustering diagram. The abscissa represents the sample, and the ordinate represents the expression of centralization and standardization. (**B**) KEGG classification column chart. The abscissa represents the proportion of genes annotated in a given pathway to the total number of annotated genes, and the ordinate represents the name of the KEGG pathway. The label on the right side of the figure represents the classification of the KEGG channel. (**C**) Wayne diagram of different genes in different groups. The non−overlapping regions of the Wayne diagram represent unique differential genes of this differential group, and overlapping regions represent common differential genes of several overlapping differential groups. (**D**) Percentage diagram of transcription factors. Different colors indicate different transcription factors, and the number after the transcription factor name represents the quantity of transcription factors.

KEGG and GO enrichment analysis of DEGs could help to elucidate the genetic differences in quinoa seeds under different developmental periods. To investigate the enrichment pattern of genes in quinoa seeds at different developmental periods, the FPKM values of genes were centralized and normalized, and K-means clustering analysis was performed. The same class of genes had similar change trends under different experimental treatments and might have similar functions, and the results assigned all 26,463 genes to four different clusters, indicating that different genes were differentially expressed at different stages with varying expression trends, which could be used as a potential marker to distinguish differential genes in quinoa seeds under different developmental periods (Figure 4A). The expression levels of FPKM values were extracted after the centralization and standardization of differential genes, hierarchical clustering analysis was performed, and the clustering heat map of each differential group was drawn (Figure S5). It could be seen that the hierarchical clustering results of differential gene expression were different during the development of *Dianli-3260* seeds. The differential genes were classified by GO, the GO term was taken as the unit, and the hypergeometric test was applied to determine whether the GO term was significantly enriched in the DEGs compared with the whole genome background. It was found that the biological process accounted for the largest proportion of the enrichment pathways and the molecular function and cellular component were less in different periods (Figure S6); moreover, the enrichment pathways of FB2 vs. RB2, FB2 vs. DB2, FB2 vs. MB2, RB2 vs. DB2, RB2 vs. MB2, and DB2 vs. MB2 can be further classified into five categories: metabolism, genetic information processing, cellular process, environmental information processing, and organismal systems. In the five categories, the metabolism category contained the largest number of pathways in all six comparison groups (Figure 4B). The degree of KEGG enrichment was measured by rich factor, Q-value, and the number of genes enriched to this pathway. Through a Venn diagram, 2626 differential genes were found in all groups: FB2 vs. RB2, FB2 vs. DB2, FB2 vs. MB2, RB2 vs. DB2, DB2 vs. MB2 had 427, 1144, 1745, 229, and 1213 differential genes, respectively (Figure 4C). It was shown that the TFs MYB, bHLH, and WD40 have important roles in flavonoid regulation: 146 MYB, 205 bHLH, and 100 WRKY TFs were detected in this study (Figure 4D), of which 433 genes are related to MYB, bHLH and MYB-related TFs (Table S12).

### 2.5. Combined Metabolomic and Transcriptomic Analysis of Flavonoid Regulation

**Figure 5:**
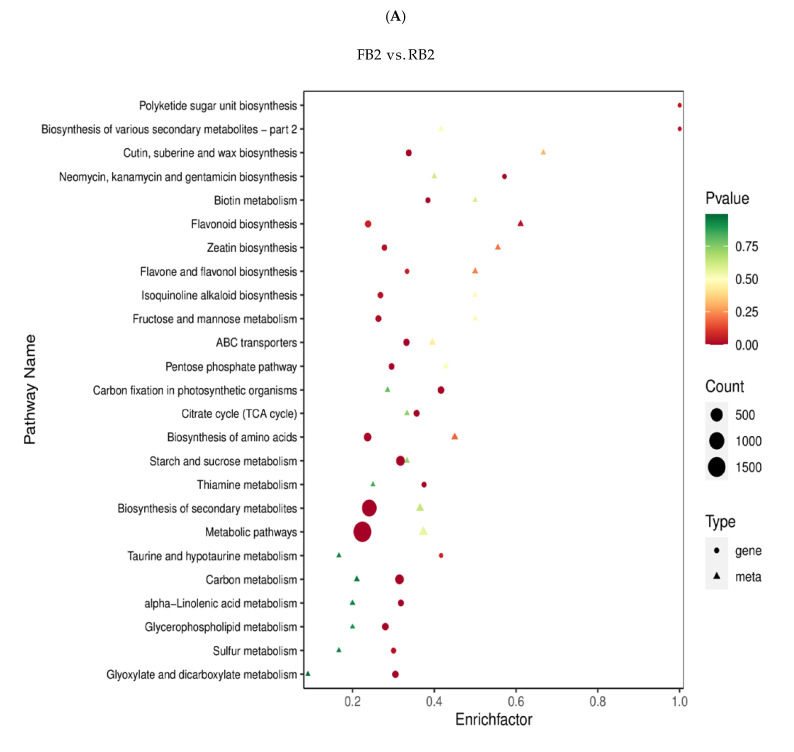

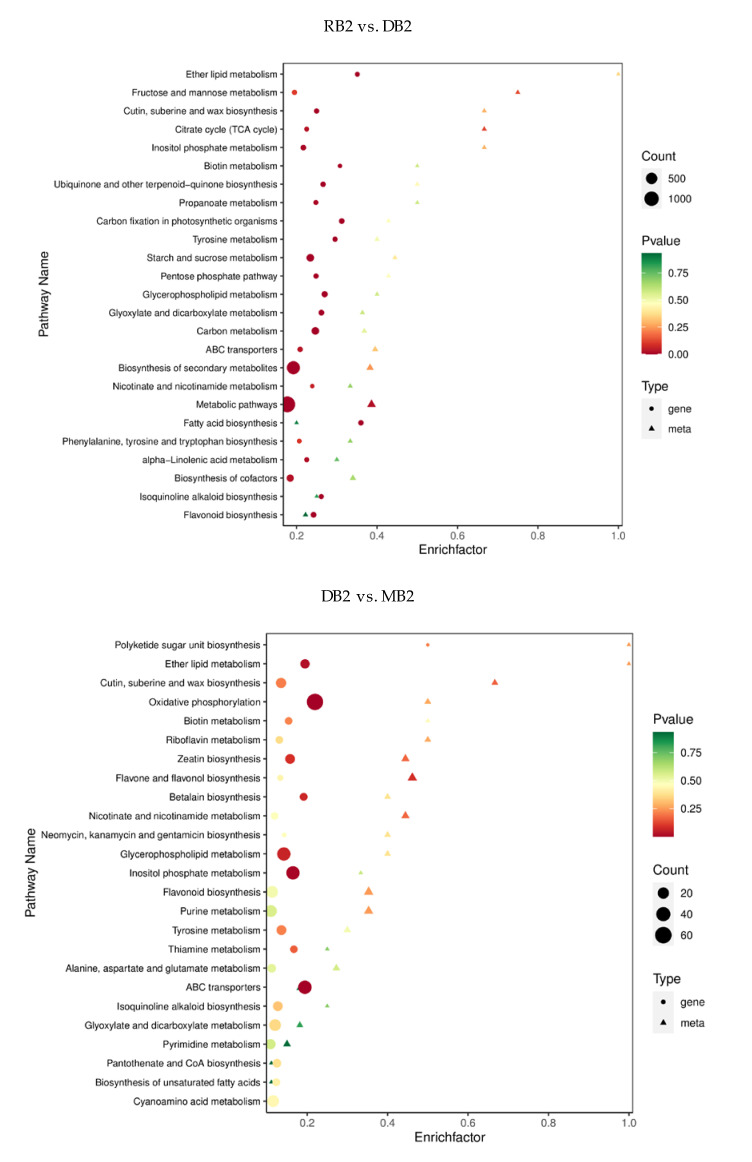

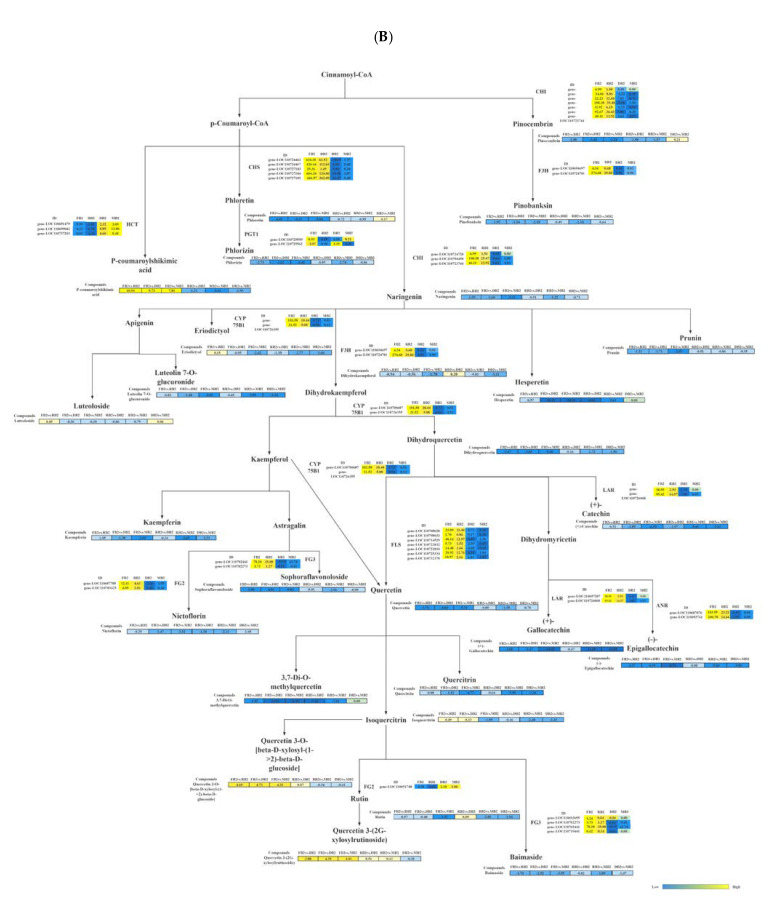
(**A**) Response mechanisms of flavonoid biosynthesis and flavone and flavonol pathways in quinoa seeds during different developmental periods. Red and green represent upregulated and downregulated genes/metabolites, respectively. (**B**) The boxes in the pathway represent differentially expressed genes (DEGs) or differentially accumulated metabolites (DAMs). CHI, chalcone isomerase; F3H, flavanone 3−hydroxylase; CHS, chalcone synthase; PGT1, phlorizin synthase; HCT, shikimate O-hydroxycinnamoyltransferase; CYP75B1, flavonoid 3′-monooxygenase; LAR, leucoanthocyanidin reductase; FLS, flavonol synthase; ANR, anthocyanidin reductase; FG2, flavonol−3−O−glucoside L−rhamnosyltransferase; FG3, flavonol−3−O−glucoside/galactoside glucosyltransferase.

To understand the differences in flavonoid synthesis in different development stages of quinoa, we integrated metabolome and transcriptome data, mapped different genes and metabolites in the same group to the KEGG pathway map simultaneously, and plotted a bar plot (Figure S7) and bubble plot (Figure 5A) according to the enrichment results. It could be seen that flavonoid contents were different in the different quinoa development stages, and the related genes and metabolites were mainly concentrated in two pathways: flavonoid biosynthesis and flavone and flavonol biosynthesis. The differential ploidy profiles of gene metabolites with Pearson correlation coefficients greater than 0.8 in each differential subgroup were shown in a nine-quadrant plot as neither gene nor metabolite differentially expressed, gene and metabolite differentially expressed in the same pattern, or gene and metabolite differentially expressed in opposite patterns (Figure S8). The results of all correlation calculations for selected differential genes and differential metabolites were plotted in a correlation clustering heat map, and the results showed that flavonoids accounted for a larger proportion (Figure S9). By comparing the flavonoid biosynthesis at four different quinoa seed development stages, we constructed the mechanism of flavonoid biosynthesis at each development stage (Figure 5B).

The results of the correlation network diagram showed that in the flavonoid biosynthetic pathway, pinocembrin, naringenin, phloretin, dihydroquercetin, quercetin, gallocatechin, and epigallocatechin had strong correlations with genes (Table 2, Figure S10). These substances showed a gradual downward trend in the four development stages of quinoa seeds; that is, the contents of FB2 were the highest and those in MB2 were the lowest. Additionally, kaempferol was one of the major flavonoids determined in quinoa seeds at different developmental stages. It connects flavonoid biosynthesis pathways with flavone and flavonol biosynthesis pathways. Notably, the content of p-coumaroylshikimic acid was the highest in RB2 and the lowest in FB2 (Figure 5B). Quercetin, nicoflorin, sophoraflavonoside, and baimaside showed strong correlations with genes in the flavone and flavonol biosynthesis pathways (Table 2, Figure S11). In addition, the quercetin 3-O-[beta-D-xylosyl-(1->2)-beta-D-glucoside] and quercetin 3-(2G-xylosylrutinoside) levels were the lowest in FB2 and higher in DB2 and MB2, and quercetin, nictoflorin, sophoraflavonoloside, and baimaside were significantly down-regulated during all four developmental periods (Figure 5B). The analysis revealed that CHI [EC:5.5.1.6] (gene-LOC110704458 and gene-LOC110723744) showed a strong positive correlation (PCC > 0.8) with pinocembrin, and the expression levels of these two genes were the highest in FB2 and showed a gradual decrease with the developmental period, which indicated that for gene-LOC110704458, gene-LOC110723744, gene LOC110704458, and gene-LOC110723744, the decrease in expression inhibited the accumulation of pinocembrin. Flavanone 3-hydroxylase [EC:1.14.11.9] (gene-LOC110724781 and gene-LOC110694697; F3H) has a strong positive correlation with pinobanksin (PCC > 0.8); gene-LOC110724781 and gene-LOC110694697 were significantly down-regulated with the development period, and the pinobanksin content gradually decreased; CHI [EC:5.5.1.6] (gene-LOC110704458, gene-LOC110734728, and gene-LOC110723744) was significantly down-regulated as the developmental period progressed, gradually inhibiting the accumulation of naringenin. In addition, chalcone synthase [EC:2.3. 1.74] (gene-LOC110724462 and gene-LOC110727183; CHS) showed a significant positive correlation with phloretin (PCC > 0.8), flavonoid 3′-monooxygenase [EC:1.14.14.82] (gene-LOC110700687 and gene-LOC110726355; CYP75B1) showed a significant positive correlation with dihydroquercetin (PCC > 0.8), flavonol synthase [EC:1.14.20.6] (gene-LOC110714529 and gene-LOC110732370; FLS) showed a significant positive correlation with quercetin (PCC > 0.8), leucoanthocyanidin reductase [EC:1.17. 1.3] (gene-LOC110697307 and gene-LOC110726068; LAR) showed a significant positive correlation with gallocatechin (PCC > 0.8), anthocyanidin reductase [EC:1.3.1.77] (gene-LOC110687076 and gene-LOC110693741; ANR) showed a significant positive correlation with epigallocatechin (PCC > 0.8), flavonol-3-O- glucoside/galactoside glucosyltransferase [EC:2.4.1.239 2.4.1.-] (gene-LOC110693695, gene-LOC110702273 and gene-LOC110702441; FG3) showed a significant positive correlation with sophoraflavonoloside (PCC > 0.8), flavonol-3-O-glucoside L-rhamnosyltransferase [EC:2.4.1.159] (gene-LOC110687785 and gene- LOC110703425; FG2) showed a significant positive correlation with nictoflorin (PCC > 0.8), and flavonol-3-O-glucoside/galactoside glucosyltransferase [EC:2.4.1.239 2.4.1.-] (gene-LOC110693695, gene-LOC110702273, gene-LOC110702441, and gene-LOC110719441; FG3) showed a significant positive correlation (PCC > 0.8) with baimaside (Table 2, Figure 5B).

RNA-Seq analysis and RT-PCR were performed on randomly selected DEGs to determine the authenticity and reliability of the transcriptome data and differential expression of the candidate genes. The RT-qPCR and RNA-Seq results were consistent for seven of the ten validated genes (gene-LOC110681848, gene-LOC110681936, gene-LOC110682196, gene-LOC110682402, gene-LOC110696170, gene-LOC110702210, and gene-LOC110702441). Hence, the transcriptome sequencing was reliable (Figure S12).

## 3. Discussion

Quinoa is rich in natural flavonoids and flavonoid compounds. The total flavonoid contents of blue- and purple-grained wheat have been found to be 212 and 96 μg/g, respectively. Similarly, the total flavonoid contents of black, blue, and purple barley have been found to be 156, 35, and 125 μg/g, respectively, and those of black and red rice are 3276 and 94 μg/g, respectively [28]. Liu found that the flavonoid content of quinoa seeds ranged from 362 to 1443 μg/g [29], indicating a relatively high flavonoid content in quinoa seeds among food grains, except for purple rice. Processing temperature and time can cause the loss of flavonoids in grains, but foods made from 100% quinoa retain most of the original flavonoid content [30]. Understanding the regulatory mechanisms of flavonoid synthesis and identifying methods to enhance the flavonoid content have long been active areas of research. However, little is known about the molecular synthesis mechanism of flavonoids in quinoa seeds at different developmental stages. Through joint analysis of transcriptomic and metabolomic changes, we examined the flavonoid biosynthesis mechanism in quinoa seeds, and verified the genes of flavonoid composition and characterization. These results deepen the current understanding of the quinoa flavonoid control network, as well as flavonoid accumulation during grain development and its related molecular mechanisms. Therefore, this study lays the foundation for future work on this aspect.

In most cases, the biosynthesis of the flavonoid backbone begins with the biosynthesis of phenylpropane initiated by cinnamoyl-CoA and p-coumaroyl-CoA [31], which further forms naringenin. Naringenin then forms dihydrokaempferol through F3H, and produces dihydroquercetin [32], owing to the LAR- and ANR-mediated formation of (+)-gallocatechin and (-)-epigallocatechin. In our study, the contents of these two substances in quinoa showed a downward trend in FB2 as the quinoa grains gradually matured; apigenin, quercetin, and kaempferol are associated with flavonoid and flavonol pathways [22]. The specific contents of flavonoids in quinoa seeds varies with the variety [26,27]. Han et al. [33] found that dark-colored quinoa seeds contain higher flavonoid contents than light-colored seeds. Liu et al. [34] found that the flavonoid contents in black and red quinoa are higher than those in yellow and white quinoa. This study found that there were also differences in the flavonoid contents of quinoa seeds at different stages of development. In total, 154 kinds of flavonoid metabolites were detected across the metabolome, mainly flavones and flavonols, which may also explain the formation of quinoa seed color. However, a previous study by Tang et al. [6] found that the pigments of red quinoa and black quinoa seeds are mainly betalains and isobetain. Therefore, further studies are needed to clarify the mechanism of quinoa seed color formation; this study provides insights into the mechanism of flavonoid biosynthesis in quinoa. Flavonoids in quinoa are generally quercetin and kaempferol; however, myricetin and isorhamnetin have been found in some varieties [35], which is consistent with this study. In the four development stages of quinoa seeds, quercetin and kaempferol, epigalocatechin, and gallocatechin have higher contents in the filling stage, and show a downward trend with the development stage. Except for p-coumaroylshimic acid, quercetin 3-O-[beta-D-xylosyl-(1->2)-beta-D-glucoside], and quercetin 3-(2G-xylosylrutinoside), the levels of most flavonoids in quinoa seeds were high in the filling period, indicating that this stage is a key period for flavonoid biosynthesis. This is in accordance with the research of Li et al. [16], who believes that the peak filling stage is the key period of flavonoid biosynthesis in Tartary buckwheat. In previous studies, the flavonoids in the leaf material of 25 Avena species were mainly identified to be glycosides [36], and the main flavonoid identified in ferox seeds was dihydroflavonoid. From the overall trend, most flavonoid metabolites show a trend of first decreasing, then increasing, and then decreasing again during seed development [37]. The flavonoid compounds in grape seeds are mainly gallic acid, catechin, and epicatechin [38], and the main flavonoid released from alfalfa seeds was identified as quercetin-3-O-galactoside [39]. In this study, quinoa flavonoids were mainly flavones and flavonols, and most of them showed a gradual downward trend during grain development. We speculate that the gradual decrease in pinocembrin content during the development period may be due to the gradual decrease in CHI expression. At the developmental stage, the gradual decrease in phloretin content may be due to the gradual decrease in CHS expression. The gradual decrease in p-coumaroylshikimic acid content during the development period may be due to the gradual decrease in HCT expression; epigalocatechin, gallocatechin, quercetin, and other substances gradually decrease during the development period. Meanwhile, it may be due to the reduced expression of corresponding structural genes, or it might be related to the reduced content of up-stream metabolites. These findings, which were different from those in other species, provide new ideas for understanding the flavonoid biosynthesis pathway of quinoa.

The biosynthesis of plant flavonoids is controlled by two types of genes. One category is structural genes, which encode enzymes in the synthesis pathway and catalyze the biosynthesis of different flavonoids in the metabolic pathway. The other category is regulatory genes, which encode TFs that regulate the spatiotemporal expression of structural genes in the synthetic pathway [40]. Genes of the early flavonoid biosynthetic pathway include PAL, C4H, 4CL, CHS, CHI, and F3H, while genes of the late flavonoid biosynthesis pathway include dihydroflavonol reductase, ANS, and ANR in the ginkgo transcriptome [41].

In this study, a variety of structural genes were also detected during the development of quinoa seeds, 22 of which were strongly correlated (>0.8) with flavonoid-related metabolites, and the expression level of these structural genes showed a downward trend with the gradual maturation of seeds, indicating that the expression level of these structural genes played a key role in the accumulation of specific flavonoid-related metabolites during the development of quinoa seeds. The key structural genes of flavonoid synthesis in plants can generally be transcriptionally regulated by TFs. These factors regulate the whole transcription process of the target gene by combining with the promoter of the target structural gene to regulate the catalytic activity of key enzymes and directly regulate the anabolic process of flavonoids [28,42]. In recent years, with the deepening of research on flavonoids, increasing numbers of related TFs have been revealed. MYB, bHLH, WD40, and other TFs play an important role in the transcriptional regulation of flavonoids [43,44,45]. The bHLH protein acts as a link between MYB and WD40 through interaction and eventually forms a stable MYB–bHLH–WD40 (MBW) ternary complex, thereby controlling the expression of flavonoid structural genes [46,47,48]. There are 434 genes associated with MYB-, bHLH-, and MYB-related TFs in this study, which can be subjected to the next step of experimentation to verify their association with flavonoid biosynthesis.

In addition, we also performed de novo gene analysis by assembling reads into transcripts using StringTie based on the positional information of reads on the matched genome. By comparing the spliced transcripts with the information of genome annotation to extract new transcripts or de novo genes, we found 50 de novo genes (Table S13); however, further studies are required to validate these findings.

## 4. Materials and Methods

### 4.1. Materials and Sample Preparation

**Figure 6:**
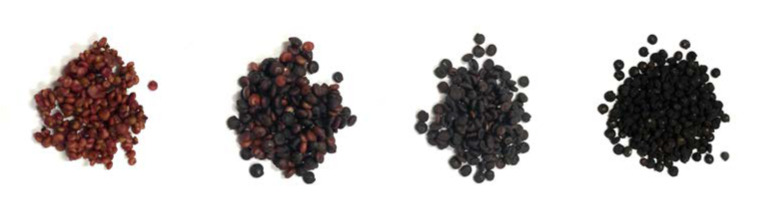
From left to right, quinoa seeds at the filling stage (FB2), milking stage (RB2), dough stage (DB2), and maturity stage (MB2).

Dianli-3260, independently selected by Yunnan Agricultural University, was planted at the Modern Agricultural Education and research base of Yunnan Agricultural University in Xundian County, Kunming (25°20′ N, 102°41′ E). Uniform and consistent seeds were selected and sown in trays (117 cm × 39 cm × 65 cm) in uniform holes, with about 20 seedlings per tray, and managed according to conventional cultivation and management techniques in the early stage (red soil: humus soil: compound fertilizer = 3:2:1, average temperature: 25.6 °C; sunshine duration: about 10 h; sowing depth: 2–3 cm). Seeds were sampled for metabolomic and transcriptomic analyses at the filling stage (FB2, 15 days after flowering), milking stage (RB2, 25 days after flowering), dough stage (DB2, 35 days after flowering), and maturity stage (MB2, 45 days after flowering) of quinoa (Wuhan Metwell Biotechnology Co., Ltd., Wuhan, China) (Figure 6), with samples for each period (12 samples in total). In order to avoid errors, we started sampling at 10 a.m. 15/25/35/45 days after the flower with an average temperature of 25.5 °C and a rainfall of 0.0 mm on the sampling day. In this experiment, we took three biological replicates and performed three technical replicates for each biological sample.

### 4.2. Extraction, Detection, and Qualitative and Quantitative Analysis of Metabolites

The quinoa seed samples in four periods were vacuum freeze-dried in a freeze dryer (SCIENTZ-100F; Ningbo Scientz Biotechnology Co., Ltd., Zhejiang, China), extracted by grinding, and then centrifuged (12,000 rpm, 10 min, 4 °C) to retrieve the supernatant and analyzed with ultraperformance liquid chromatography-tandem mass spectrometry (UPLC-MS/MS). The data acquisition instrument system comprises an ultraperformance liquid chromatograph (Nexera X2; Shimadzu, Kyoto, Japan) used with tandem mass spectrometry (MS) (QTRAP^®^ 4500 LC-MS/MS System; Applied Biosystems, Waltham, MA, USA).

A triple quadrupole linear ion trap mass spectrometer (QTRAP) [49] was used for mass spectrometry. Analyst software v.6.3 (AB Sciex, Toronto, Canada) was used to regulate the positive and negative ion modes, while ion source gas I (GSI), gas II (GSII), and curtain gas (CUR) were set to 50 psi, 60 psi, and 25.0 psi, respectively. Triple quadrupole (QQQ) scans were performed using multiple reaction monitoring (MRM) mode with the collision gas (nitrogen) set to medium. Specific MRM ion pairs were monitored based on the eluted metabolites. The metware database (MWDB) was used to identify each analyte detected by secondary MS. The obtained spectra were used for metabolite profiling, peak area integration, and integration correction [50]. Quality control (QC) samples were prepared by mixing sample extracts. Reproducibility was monitored by analyzing one QC sample for every 10 experimental samples. Metabolite extraction and detection accuracy was determined by overlapping the total ion flow diagrams from several QC samples [51,52]. Using multivariate statistical analysis to maximize the retention of raw data, the data were simplified and downscaled to create numerical models using the prcomp function in R software (v.3.5.1, https://www.r-project.org/,accessed on 17 February 2022) [53,54]. Heatmaps were drawn using the pheatmap R package (v.1.0.12, https://jokergoo.github.io/ComplexHeatmap-reference/book/, accessed on 17 February 2022). Metabolites in different samples were analyzed by hierarchical clustering. Orthogonal partial least squares discriminant analysis (OPLS-DA) was used to extract the components in the independent variable X and the dependent variable Y and were used to screen differential variables [52,55]. Based on the OPLS-DA results, the variable importance in projection was combined with the *p*-values and fold changes to further screen differential metabolites [55]. The metabolites were considered significant if they differed more than 2-fold or less than 0.5 between the control and treated groups. The relative contents of all differential metabolites are standardized by the Z-score, followed by K-means cluster analysis, which can be used to analyze the change trends of the relative contents of metabolites in different groups. After screening the differential metabolites, they were annotated using the Kyoto Encyclopedia of Genes and Genomes (KEGG) database (https://www.kegg.jp/kegg/compound/, accessed on 17 February 2022) [56] and their significance was determined using hypergeometric tests.

### 4.3. Transcriptome Sequencing and Data Analysis

RNA extraction, RNA detection, library construction, sequencing, and bioinformatic analysis were performed by Wuhan Metaville Biotechnology Co., Ltd. (www.metware.cn. Wuhan, China, accessed on 15 February 2022). After library construction, initial quantification was performed with a Qubit 2.0 Fluorometer (Life Technologies, Carlsbad, CA, USA, followed by detection of the library insert size using a 2100 Bioanalyzer (Agilent, Santa Clara, CA, USA). After the insert size met expectations, qRT-PCR was used to accurately quantify the effective concentration of the library (the effective concentration of the library was higher than 2 nM) to ensure the quality of the library. The reaction system set in the experiment was 20 µL, comprising 2× Perfectstart^TM^ SYBR qPCR Supermix (10 µL), calibration solution (0.4 µL), nuclease free water (5.8 µL), 0.4 µL of each 10 mM primer, cDNA (3 μL; 200 μg/μL). The thermal cycle setting steps in the experiment are as follows: 94 °C (30 s), 94 °C (5 s), 40 cycles, 60 °C (30 s). RT- qPCR was performed to accurately quantify the effective library concentration. The library was qualified and used for sequencing on a HiSeq platform (Illumina, San Diego, CA, USA). The data were filtered to obtain clean data and compared with the reference genome (https://www.ncbi.nlm.nih.gov/genome/?Term=Chenopodium+quinoa+Willd, accessed on 15 February 2022). Genes were aligned using the HISAT (v.2.1.0, https://daehwankimlab.github.io/hisat2/, accessed on 16 February 2022) and Bowtie 2 (v.2.4.4, http://bowtie-bio.sourceforge.net/bowtie2/manual.shtml, accessed on 16 February 2022) software programs [57,58]. BLASTX (v.2.7.1, https://ftp.ncbi.nlm.nih.gov/blast/executables/blast+/2.7.1/, accessed on 16 February 2022) was used to compare the new gene with KEGG, Gene Ontology (GO), National Center for Biotechnology Information (NCBI) non-redundant (NR), Swiss-Prot, EuKaryotic Orthologous Groups (KOG), and TrEMBL database sequences to obtain the annotation results, and the expressed values of all genes were calculated and normalized to fragments per kilobase of transcript per million fragments mapped (FPKM). Differential expression analysis was performed using DESeq2 (v.1.22.2, https://bioconductor.org/packages/release/bioc/html/DESeq2.html, accessed on 16 February 2022) [59], the input data were the original readcount file of genes, and the standardization method was provided by DESeq software The false discovery rate was obtained by multiple hypothesis testing correction using the Benjamini–Hochberg method. The final differential gene criteria were |log2Fold Change| ≥ 1 and false discovery rate <0.05 [60]. We used the ‘ggplot2′ R package (https://cran.r-project.org/web/packages/ggplot2/index.html, accessed on 16 February 2022) to draw the box plot. The input data are the FPKM values of the gene in each sample (the logarithm of FPKM with a base of 10 will be taken during the drawing). After screening differential genes, the original counts were filtered directly according to the expression volume, and compared with KEGG, GO, NR, Swiss-Prot, KOG, Pfam, and TrEMBL databases by using BLASTX (v.2.7.1, https://ftp.ncbi.nlm.nih.gov/blast/executables/blast+/2.7.1/, accessed on 17 February 2022) software, and the amino acid sequences were compared with Pfam database using Hmmer software to obtain the annotation information of seven databases of transcripts. Pathway significance enrichment analysis takes the pathway in the KEGG database as the unit and applies a hypergeometric test to find out the pathways that are significantly enriched in DEGs compared with the whole genome background. The formula for calculating hypergeometric distribution is as follows:

(1) $$P=1-\sum_{i=0}^{m-1}\frac{\left(\begin{array}{c} M\\ i \end{array}\right)\left(\begin{array}{c} N-M\\ n-i \end{array}\right)}{\left(\begin{array}{c} N\\ n \end{array}\right)}$$

where *N* represents the number of genes with KEGG annotation in all genes, *n* represents the number of differential genes in *N*, *M* represents the number of genes in a KEGG pathway in *N*, and *m* represents the number of differential genes in a KEGG pathway in *M*.

### 4.4. Combined Transcriptome and Metabolome Analysis

Based on the differential metabolite and gene analysis results, differential genes and metabolites from the same treatment group were mapped to KEGG pathway maps to investigate the relationship between genes and metabolites. Histograms were drawn to demonstrate the degree of difference in metabolite and pathway enrichment. Correlation analysis was performed for genes and metabolites detected in each differential subgroup. Pearson correlation coefficients for genes and metabolites were calculated using the ‘cor’ program in R (v.1.9.12.31, https://igraph.org/, accessed on 25 February 2022). Quadrant plots were used to show the differential multiplicity of genes and metabolites with Pearson correlation coefficients greater than 0.8 in each differential subgroup. All differential genes and metabolites were selected to build the Two-way Orthogonal Partial Least Squares (O2PLS) model with the R package ‘OmicsPLS’ (v.1.2.0, https://rdrr.io/cran/OmicsPLS/, accessed on 25 February 2022). Variables with higher correlations and weights were initially determined by loading plots to identify the important variables affecting another cohort [61].

### 4.5. RT-qPCR

RNA extracted from quinoa seeds was used for RT-qPCR, which was performed in triplicate. The PCR primers were designed with BeaconDesign v. 7.9 (https://beacon-designer.software.informer.com/7.9/, accessed on 22 July 2022) (Table S14). TUB-1 was used as an internal reference gene. PCR was performed on an ABI Prism 7500 (Applied Biosystems, Foster City, CA, USA) using PerfectStartTM SYBR qPCR Supermix reagent (TransGen Biotech, Beijing, China). Normalized expression of each sample was analyzed using the 2^−ΔΔCt^ method [62].

## 5. Conclusions

In this study, we explored the differences in flavonoid accumulation and the differentially expressed structural genes and TFs involved in flavonoid synthesis during the four developmental stages of quinoa seeds. We also verified some of the DEGs by RT-qPCR, clarifying the regulatory mechanism of flavonoid biosynthesis at the different developmental stages. Through the combined analysis of the transcriptome and metabolome, 154 flavonoid-related metabolites were detected in four development stages of quinoa seeds, and 39,738 genes were annotated with KEGG functions. The biosynthetic regulation mechanism of quinoa seed flavonoids was mainly concentrated in two pathways: the flavonoid biosynthesis and flavone and flavonol pathways. Correlation analysis between differential flavonoid metabolites and transcriptome changes during quinoa grain development showed that the correlation between 11 differential flavonoid metabolites and 22 structural genes was greater than 0.8, which were key factors for the difference in flavonoid biosynthesis during different quinoa grain development stages. We believe that the findings of this study elucidate flavonoid composition and accumulation patterns and the molecular mechanism of flavonoid biosynthesis during quinoa grain development, offering important information to guide the development of quinoa products.

## Figures and Tables

**Table 1:** Statistical table of different genes in different groups.

Compared Samples	Total number of DEGs with Significant Difference	Total Number of DEGs Significantly Up-Regulated	Total Number of DEGs Significantly Down-Regulated
DB2_vs_MB2	6671	2638	4033
FB2_vs_DB2	17,282	7986	9296
FB2_vs_MB2	20,231	8398	11,833
FB2_vs_RB2	11,307	5900	5407
RB2_vs_DB2	8609	2916	5693
RB2_vs_MB2	15,107	5957	9150

**Table 2:** Correlation analysis of differential metabolites and differential genes.

KEGG Pathway	Gene Name	KEGG	Meta Name	Compounds	PCC
Flavonoid biosynthesis	Gene-LOC110704458	K01859 [EC:5.5.1.6]	MWSHY0124	Pinocembrin	0.9025
	gene-LOC110723744	K01859 [EC:5.5.1.6]	MWSHY0124	Pinocembrin	0.8990
	gene-LOC110724781	K00475 [EC:1.14.11.9]	mws0914	Pinobanksin	0.9714
	gene-LOC110694697	K00475 [EC:1.14.11.9]	mws0914	Pinobanksin	0.8201
	gene-LOC110704458	K01859 [EC:5.5.1.6]	pme0376	Naringenin	0.9580
	gene-LOC110734728	K01859 [EC:5.5.1.6]	pme0376	Naringenin	0.8333
	gene-LOC110723744	K01859 [EC:5.5.1.6]	pme0376	Naringenin	0.8695
	gene-LOC110724462	K00660[EC:2.3.1.74]	pme1201	Phloretin	0.8160
	gene-LOC110727183	K00660[EC:2.3.1.74]	pme1201	Phloretin	0.8081
	gene-LOC110700687	K05280 [EC:1.14.14.82]	mws0044	Dihydroquercetin	0.8936
	gene-LOC110726355	K05280 [EC:1.14.14.82]	mws0044	Dihydroquercetin	0.8943
	gene-LOC110714529	K05278 [EC:1.14.20.6]	pme2954	Quercetin	0.8902
	gene-LOC110732370	K05278 [EC:1.14.20.6]	pme2954	Quercetin	0.9283
	gene-LOC110697307	K13081 [EC:1.17.1.3]	mws0049	Gallocatechin	0.9966
	gene-LOC110726068	K13081 [EC:1.17.1.3]	mws0049	Gallocatechin	0.9270
	gene-LOC110687076	K08695 [EC:1.3.1.77]	mws0042	Epigallocatechin	0.9000
	gene-LOC110693741	K08695 [EC:1.3.1.77]	mws0042	Epigallocatechin	0.9052
Flavone and flavonol biosynthesis	gene-LOC110693695	K22794 [EC:2.4.1.239 2.4.1.-]	Lmyn001269	Sophoraflavonoloside	0.8397
	gene-LOC110702273	K22794 [EC:2.4.1.239 2.4.1.-]	Lmyn001269	Sophoraflavonoloside	0.8597
	gene-LOC110702441	K22794 [EC:2.4.1.239 2.4.1.-]	Lmyn001269	Sophoraflavonoloside	0.9534
	gene-LOC110687785	K22772 [EC:2.4.1.159]	MWSHY0050	Nictoflorin	0.8557
	gene-LOC110703425	K22772 [EC:2.4.1.159]	MWSHY0050	Nictoflorin	0.9468
	gene-LOC110693695	K22794 [EC:2.4.1.239 2.4.1.-]	MWSHY0162	Baimaside	0.9084
	gene-LOC110702273	K22794 [EC:2.4.1.239 2.4.1.-]	MWSHY0162	Baimaside	0.8454
	gene-LOC110702441	K22794 [EC:2.4.1.239 2.4.1.-]	MWSHY0162	Baimaside	0.9706
	gene-LOC110719441	K22794 [EC:2.4.1.239 2.4.1.-]	MWSHY0162	Baimaside	0.8579
	gene-LOC110700687	K05280 [EC:1.14.14.82]	pme2954	Quercetin	0.8817
	gene-LOC110693695	K22794 [EC:2.4.1.239 2.4.1.-]	pme2954	Quercetin	0.8290
	gene-LOC110726355	K05280 [EC:1.14.14.82]	pme2954	Quercetin	0.8807

## Data Availability

Not applicable.

## Supplementary Materials

The following supporting information can be downloaded at: https://www.mdpi.com/article/10.3390/metabo12100887/s1, Figure S1: Schematic diagram of mass spectrometry multi reaction monitoring mode; Figure S2: Total Ion Current Diagram of Mass Spectrometry Analysis of Mixed Samples; Figure S3: OPLS-DA score chart; Figure S4: OPLS-DA model validation diagram; Figure S5: Differential gene clustering heat map; Figure S6: Column diagram of differential gene GO enrichment; Figure S7: KEGG enrichment analysis bar chart; Figure S8: Correlation analysis and nine-quadrant chart; Figure S9: Correlation Clustering heat map; Figure S10: Correlation network diagram of flavonoid biosynthesis pathway; Figure S11: Correlation network diagram of flavone and flavonol biosynthesis pathways; Figure S12: Comparison of RT-qPCR and RNA-Seq results. Table S1: Flavonoids related metabolites during grain development of quinoa; Table S2: Number and classification of the detected flavonoid metabolites are shown for FB2 vs. RB2; Table S3: Number and classification of the detected flavonoid metabolites are shown for FB2 vs. DB2; Table S4: Number and classification of the detected flavonoid metabolites are shown for FB2 vs. MB2; Table S5: Number and classification of the detected flavonoid metabolites are shown for RB2 vs. DB2; Table S6: Number and classification of the detected flavonoid metabolites are shown for RB2 vs. MB2; Table S7: Number and classification of the detected flavonoid metabolites are shown for DB2 vs. MB2; Table S8: K-means clustering specific metabolite information; Table S9: Specific metabolite information of differential Wayne diagram; Table S10: Quality_control of all sample summary; Table S11: The comparison efficiency; Table S12: Detected gene TF; Table S13: De novo genes; Table S14: PCR primers.
