# Supplementary material for: Integrative Analysis of the Metabolome and Transcriptome Provides Insights into the Mechanisms of Flavonoid Biosynthesis in Quinoa Seeds at Different Developmental Stages
Source: Metabolites. 2022 Sep 22;12(10):887. doi: 10.3390/metabo12100887 (PMC9609036; doi:10.3390/metabo12100887)
Supplement: Supplementary file 1 [file metabolites-12-00887-s001.zip › Figure.S11.pdf]

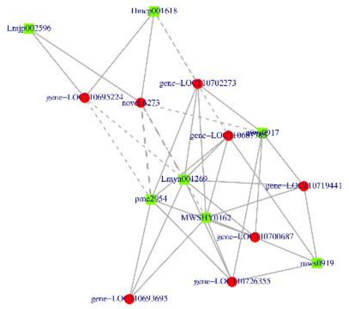

FB2 VS. RB2

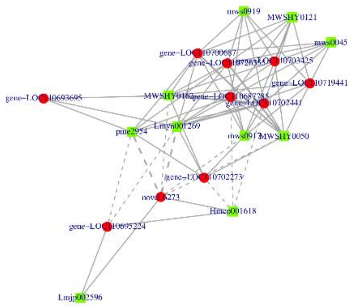

FB2 VS. DB2

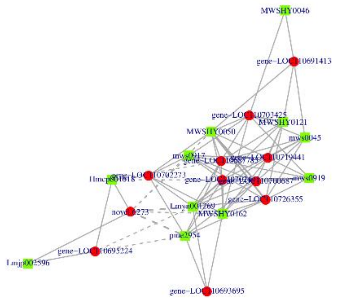

FB2 VS. MB2

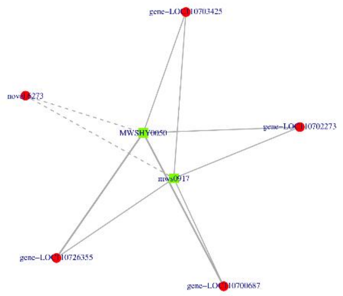

RB2 VS. DB2

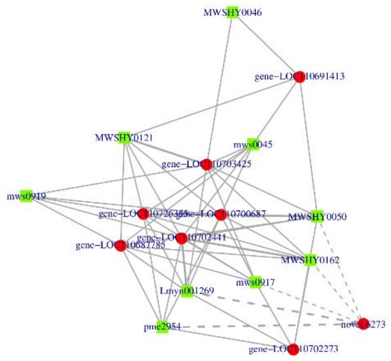

RB2 VS. MB2

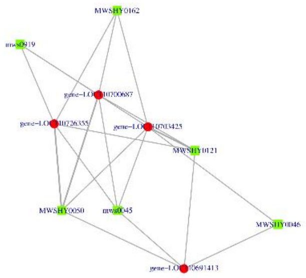

DB2 VS. MB2

Figure S11. Correlation network diagram of flavone and flavonol biosynthesis pathways. Note: the metabolites in the figure are marked with green squares and the genes are marked with red circles. The solid line represents positive correlation and the dotted line represents negative correlation
