# Supplementary material for: Integrative Analysis of the Metabolome and Transcriptome Provides Insights into the Mechanisms of Flavonoid Biosynthesis in Quinoa Seeds at Different Developmental Stages
Source: Metabolites. 2022 Sep 22;12(10):887. doi: 10.3390/metabo12100887 (PMC9609036; doi:10.3390/metabo12100887)
Supplement: Supplementary file 1 [file metabolites-12-00887-s001.zip › Figure.S12.pdf]

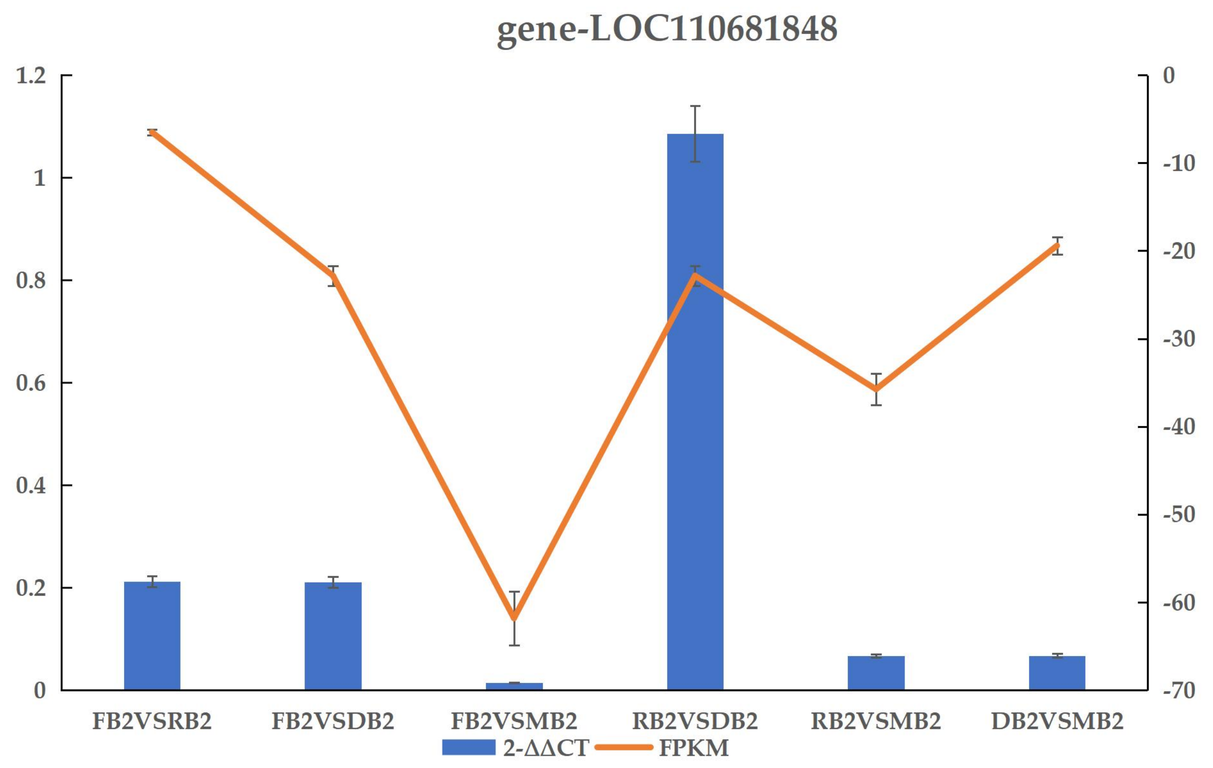
gene-LOC110681848
1.2
1
0.8
0.6
0.4
0.2
0
0
-10
-20
-30
-40
-50
-60
-70
FB2VSRB2
FB2VSDB2
FB2VSMB2
RB2VSDB2
RB2VSMB2
DB2VSMB2
2-ΔΔCT
FPKM

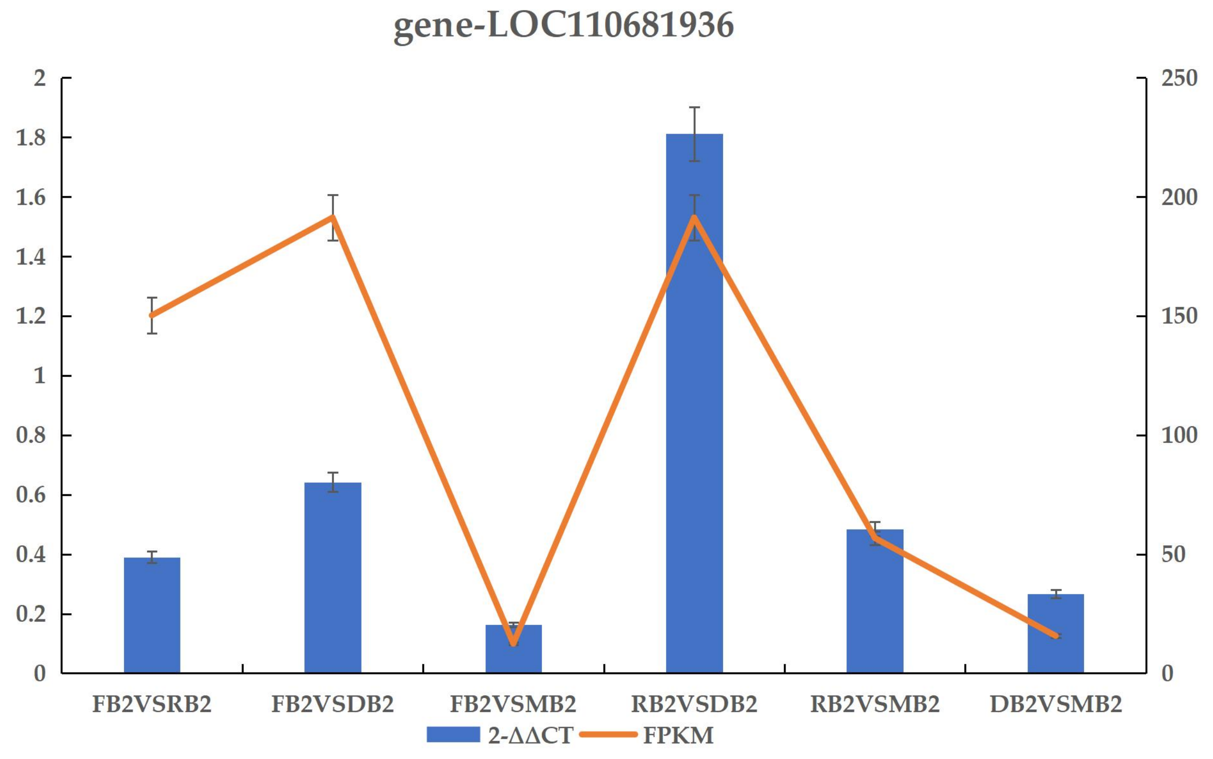
gene-LOC110681936
2
1.8
1.6
1.4
1.2
1
0.8
0.6
0.4
0.2
0
250
200
150
100
50
0
FB2VSRB2
FB2VSDB2
FB2VSMB2
RB2VSDB2
RB2VSMB2
DB2VSMB2
2-ΔΔCT
FPKM

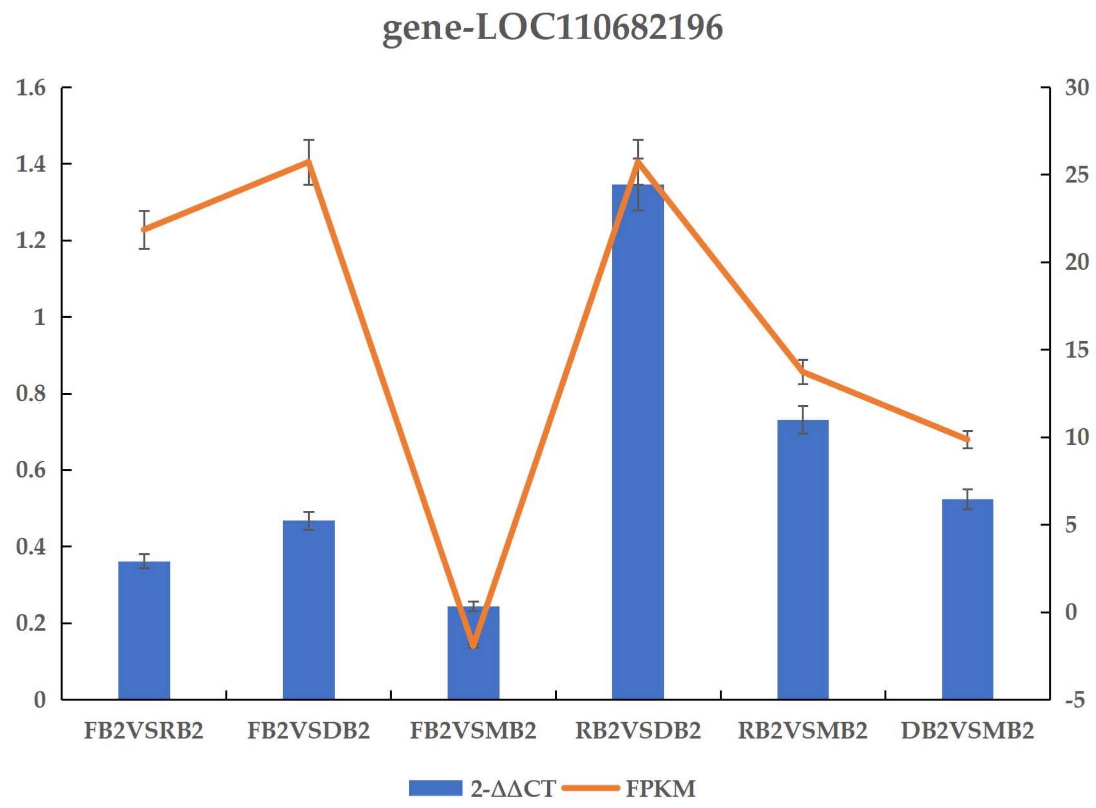
gene-LOC110682196
1.6
1.4
1.2
1
0.8
0.6
0.4
0.2
0
30
25
20
15
10
5
0
-5
FB2VSRB2
FB2VSDB2
FB2VSMB2
RB2VSDB2
RB2VSMB2
DB2VSMB2
2-ΔΔCT
FPKM

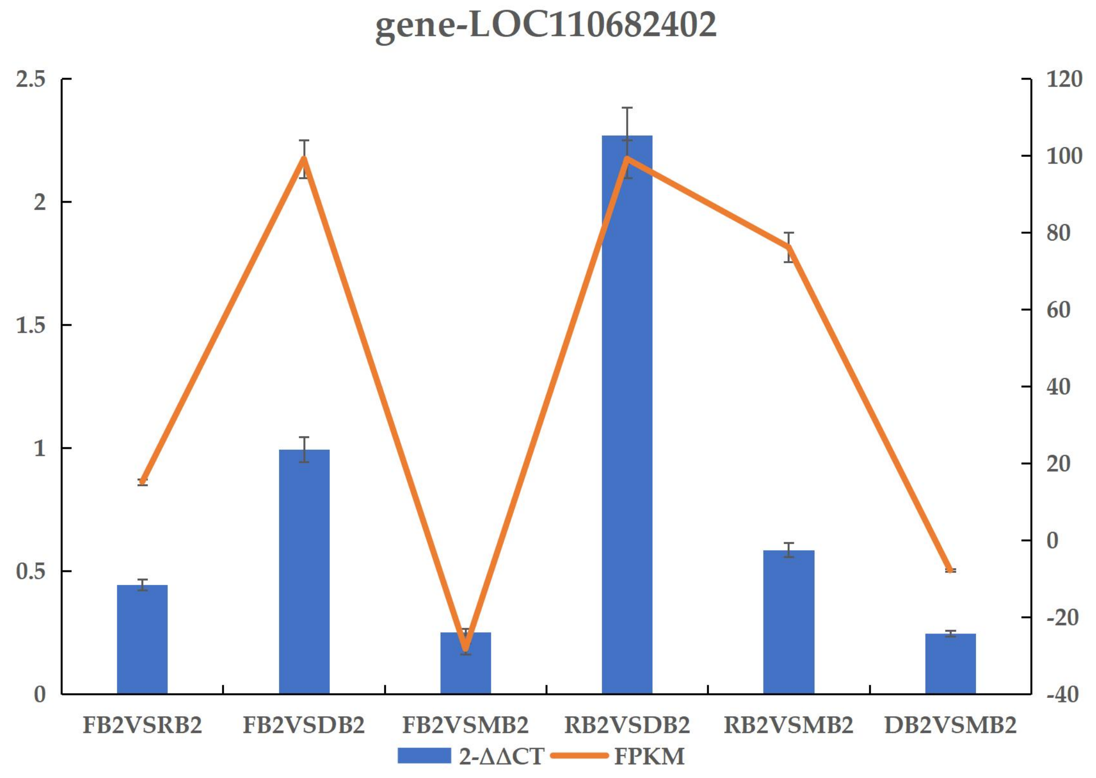
gene-LOC110682402
2.5
2
1.5
1
0.5
0
120
100
80
60
40
20
0
-20
-40
FB2VSRB2
FB2VSDB2
FB2VSMB2
RB2VSDB2
RB2VSMB2
DB2VSMB2
2-ΔΔCT
FPKM

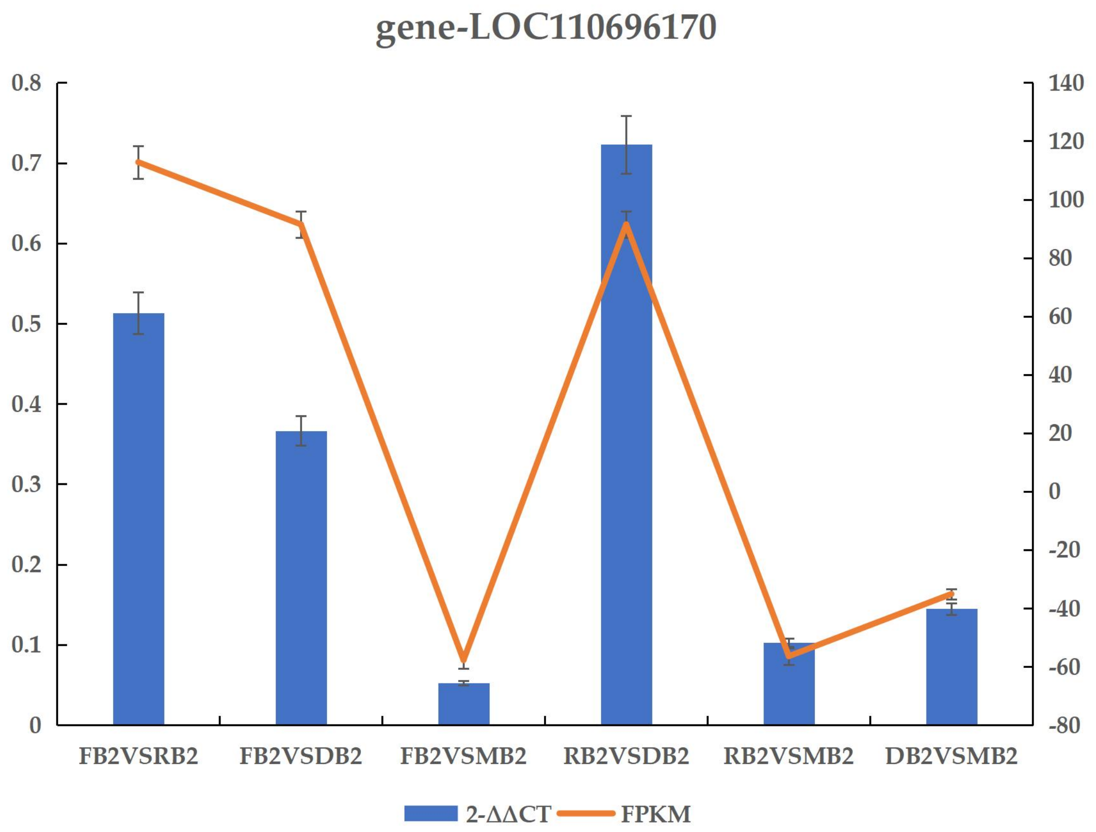
gene-LOC110696170
0.8
0.7
0.6
0.5
0.4
0.3
0.2
0.1
0
140
120
100
80
60
40
20
0
-20
-40
-60
-80
FB2VSRB2
FB2VSDB2
FB2VSMB2
RB2VSDB2
RB2VSMB2
DB2VSMB2
2-ΔΔCT
FPKM

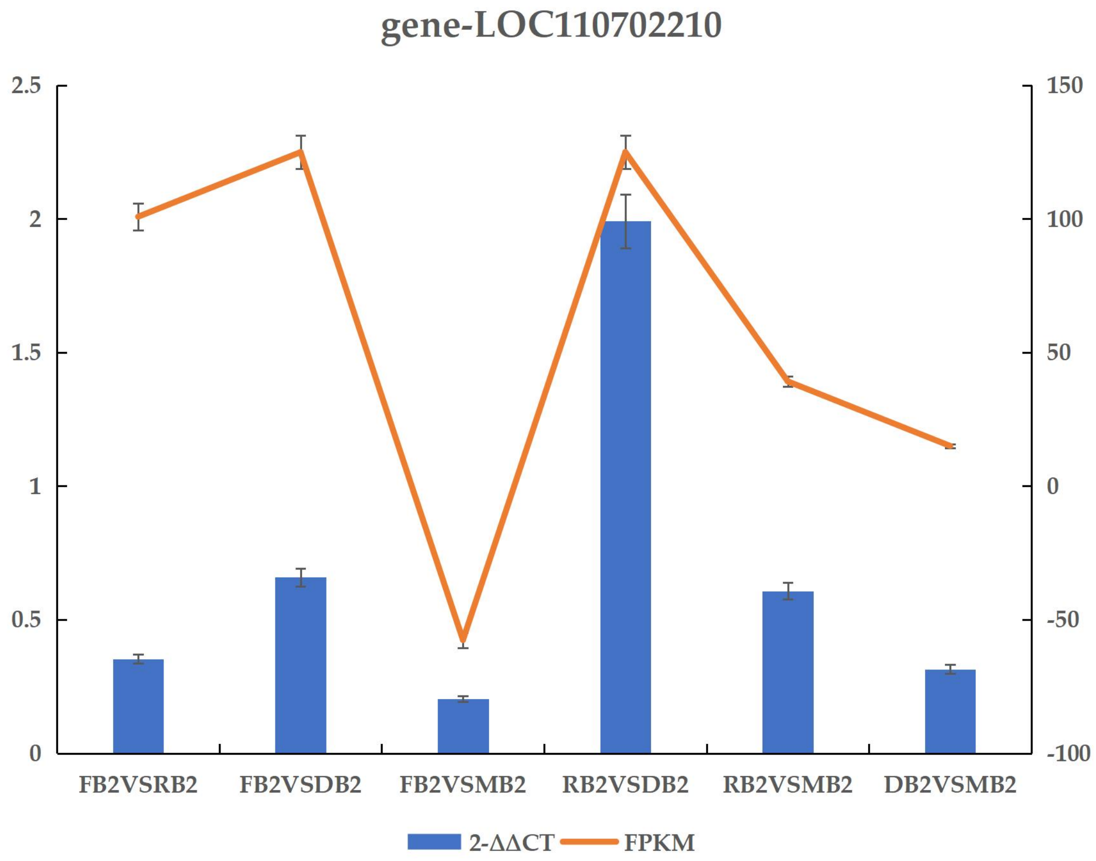
gene-LOC110702210
2.5
2
1.5
1
0.5
0
150
100
50
0
-50
-100
FB2VSRB2
FB2VSDB2
FB2VSMB2
RB2VSDB2
RB2VSMB2
DB2VSMB2
2-ΔΔCT
FPKM

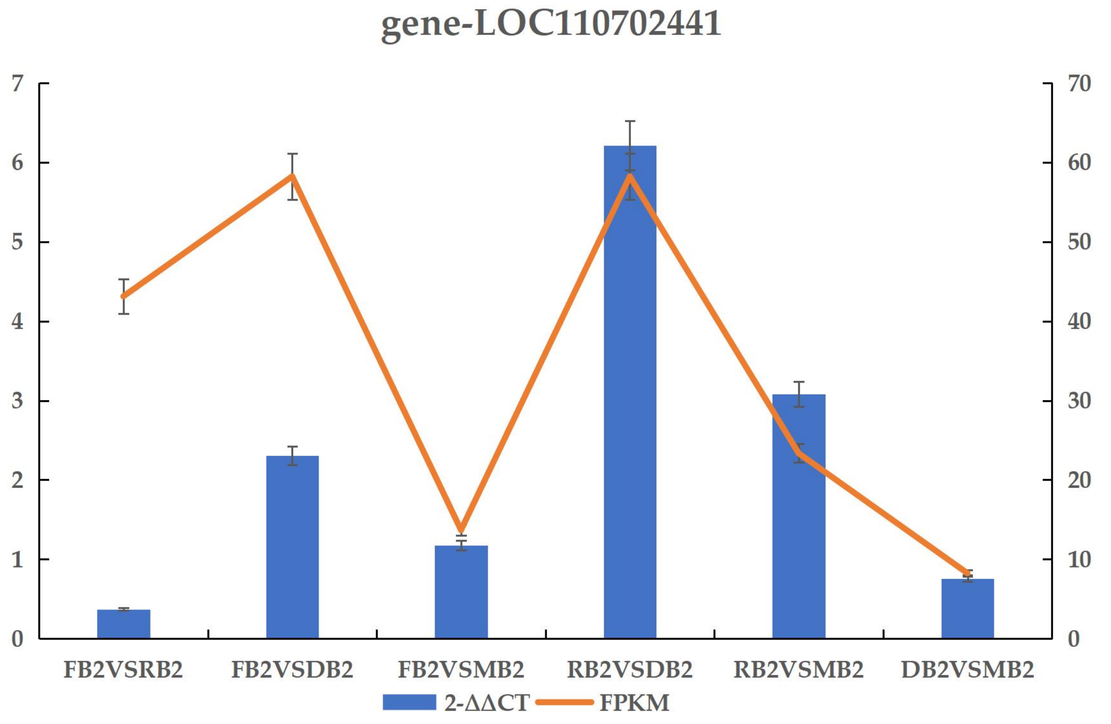

Figure S12. Comparison of RT-qPCR and RNA-Seq results. Histogram: RT-qPCR results. Broken-line diagram: RNA-Seq data. The ordinate represents the expression level. The abscissa represents different groups.
