# Supplementary material for: Integrative Analysis of the Metabolome and Transcriptome Provides Insights into the Mechanisms of Flavonoid Biosynthesis in Quinoa Seeds at Different Developmental Stages
Source: Metabolites. 2022 Sep 22;12(10):887. doi: 10.3390/metabo12100887 (PMC9609036; doi:10.3390/metabo12100887)
Supplement: Supplementary file 1 [file metabolites-12-00887-s001.zip › Figure.S3.pdf]

FB2 vs. RB2

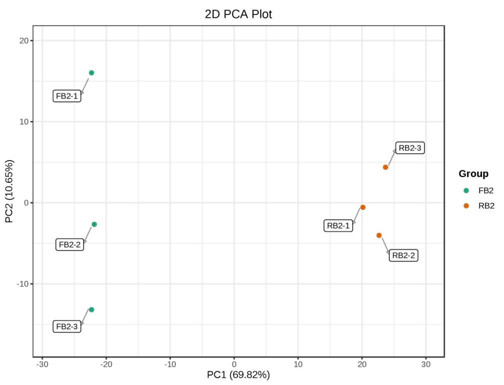

FB2 vs. DB2

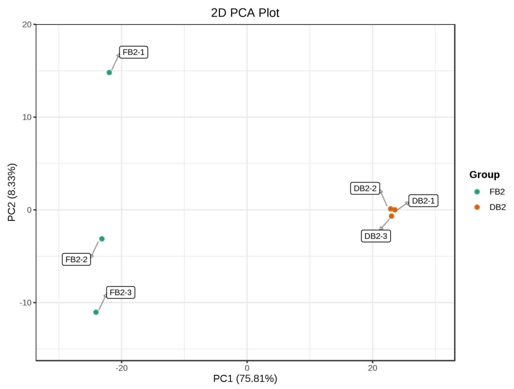

FB2 vs. MB2

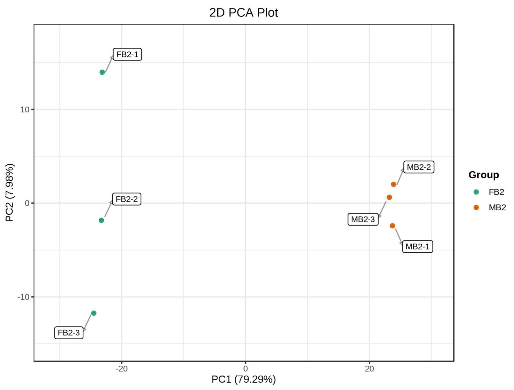

RB2 vs. DB2

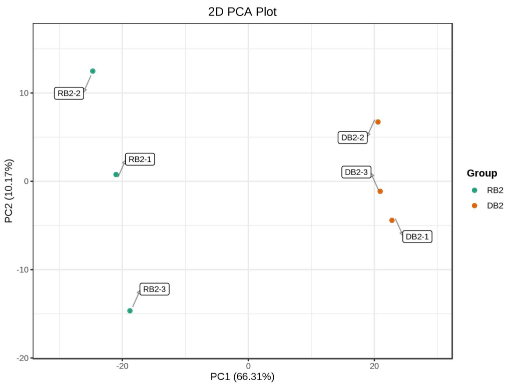

RB2 vs. MB2

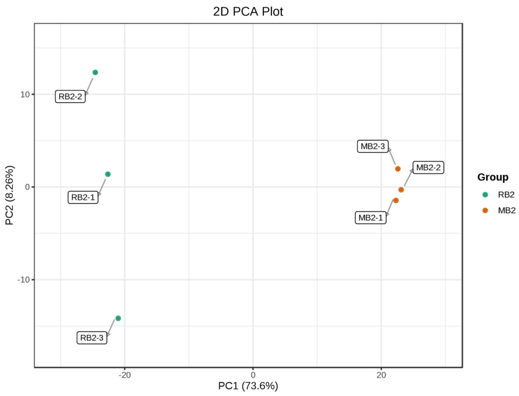

DB2 vs. MB2

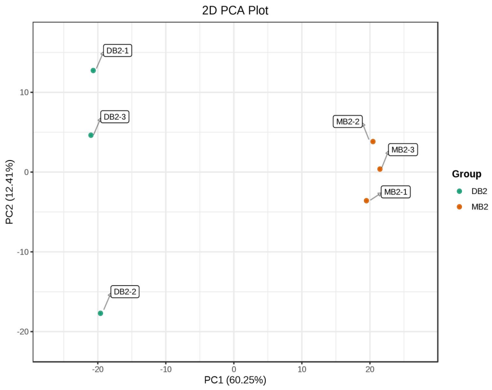

Figure S3. OPLS-DA score chart. Note: the abscissa represents the predicted PC. The differences between groups are in the direction of the abscissa. The ordinate represents the orthogonal PC. The differences within groups are in the direction of the ordinate. The percentage represents the interpretation of the PC to the dataset. Each point in the figure represents a sample. Samples in the same group are represented by the same color. Each group is a unique variety.
