# Supplementary material for: Integrative Analysis of the Metabolome and Transcriptome Provides Insights into the Mechanisms of Flavonoid Biosynthesis in Quinoa Seeds at Different Developmental Stages
Source: Metabolites. 2022 Sep 22;12(10):887. doi: 10.3390/metabo12100887 (PMC9609036; doi:10.3390/metabo12100887)
Supplement: Supplementary file 1 [file metabolites-12-00887-s001.zip › Figure.S4.pdf]

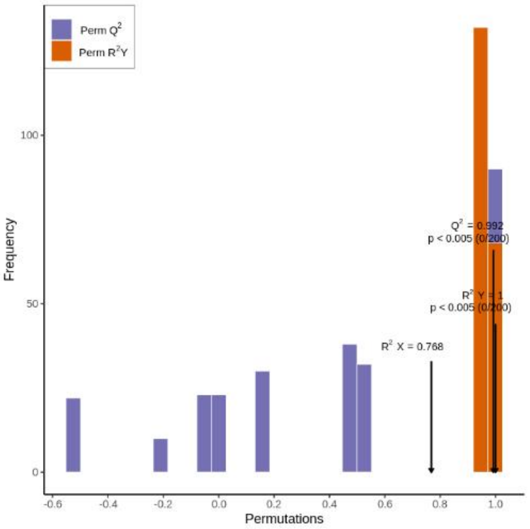

FB2 VS. RB2

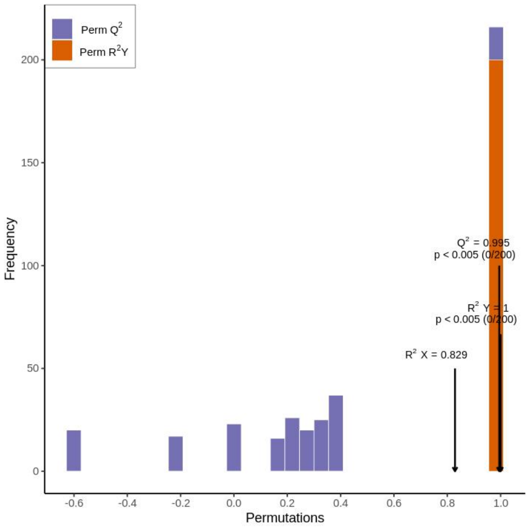

FB2 VS. DB2

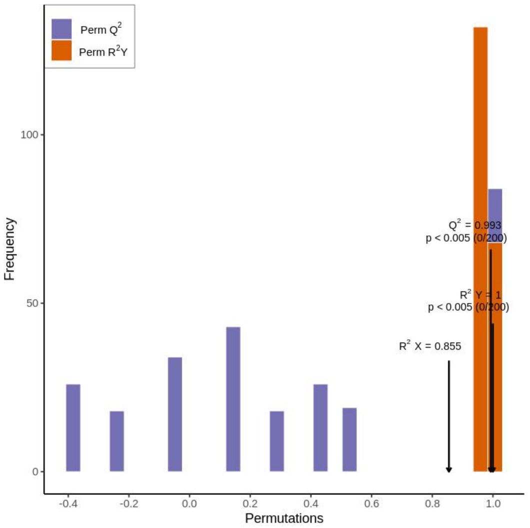

FB2 VS. MB2

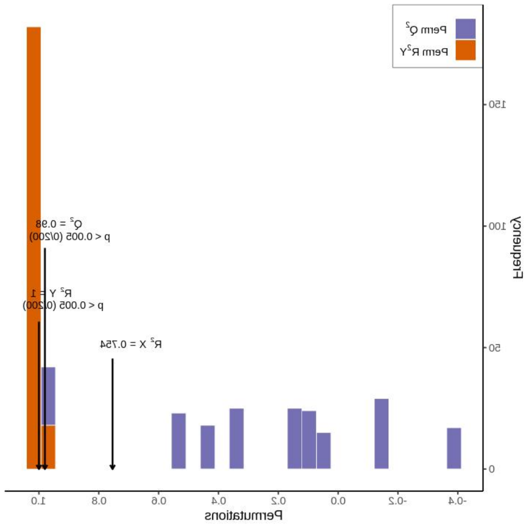

RB2 VS. DB2

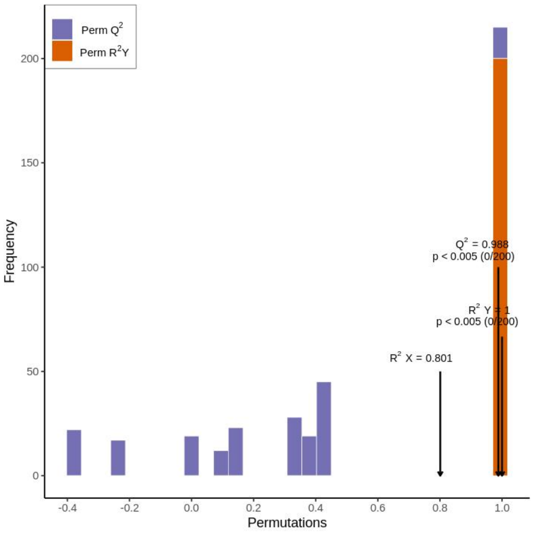

RB2 VS. MB2

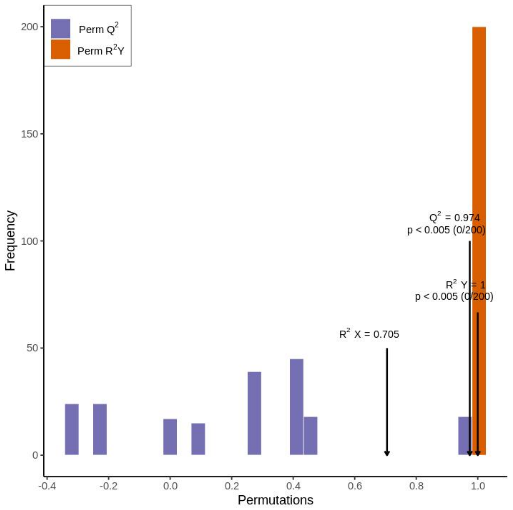

DB2 VS. MB2

Figure S4. OPLS-DA model validation diagram. The abscissa represents the model accuracy. The ordinate is the model classification effect frequency. The model performs 200 random arrangements and combination experiments on the data. If P = 0.02 for Q2, the prediction ability of four random grouping models in this permutation test is better than that of the OPLS-DA model. If P = 0.545 for R2Y, there are 109 random grouping models in the permutation test and their rate of interpretation for the Y matrix is better than that of the OPLS-DA model. In general, the model is optimal at $P < 0.05$.
