# Supplementary material for: Integrative Analysis of the Metabolome and Transcriptome Provides Insights into the Mechanisms of Flavonoid Biosynthesis in Quinoa Seeds at Different Developmental Stages
Source: Metabolites. 2022 Sep 22;12(10):887. doi: 10.3390/metabo12100887 (PMC9609036; doi:10.3390/metabo12100887)
Supplement: Supplementary file 1 [file metabolites-12-00887-s001.zip › Figure.S5.pdf]

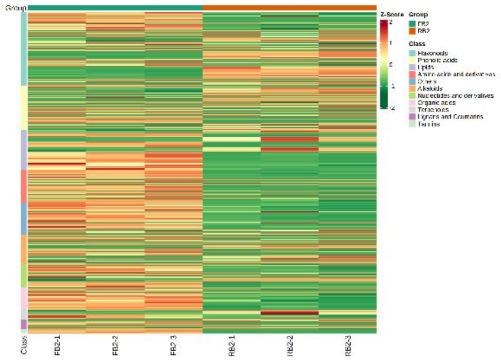

FB2 VS. RB2

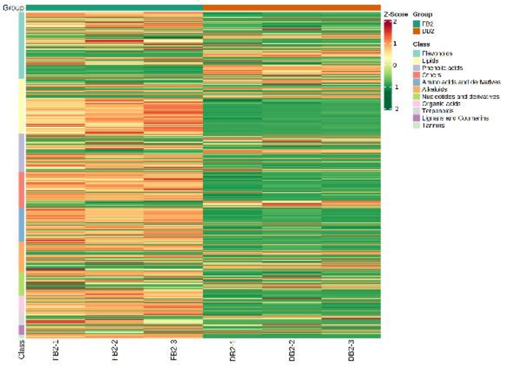

FB2 VS. DB2

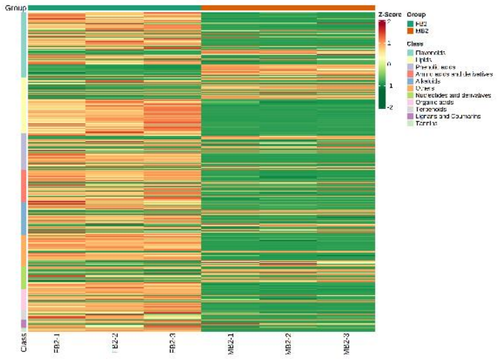

FB2 VS. MB2

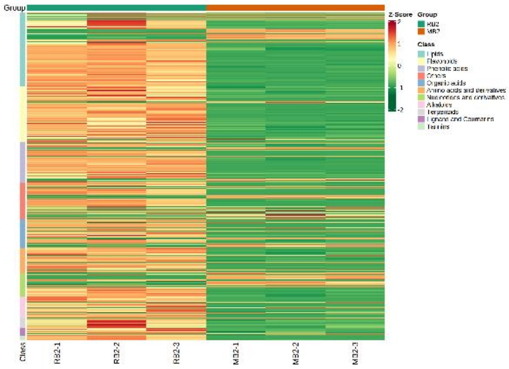

RB2 VS. DB2

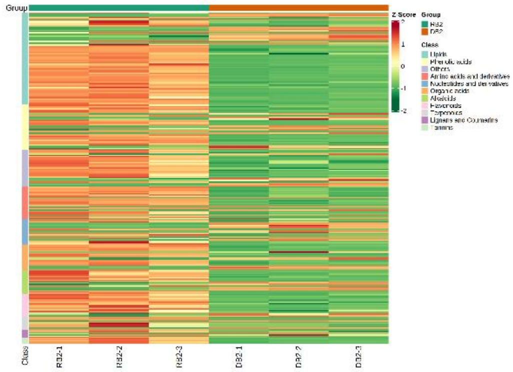

RB2 VS. MB2

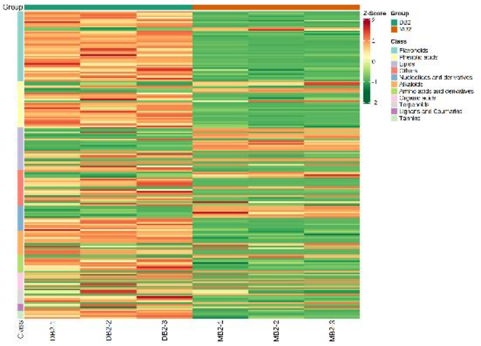

DB2 VS. MB2

Figure S5. Differential gene clustering heat map. The abscissa represents the sample name and hierarchical clustering results, and the ordinate represents the differential genes and hierarchical clustering results. Red indicates high expression and green indicates low expression.
