# Supplementary material for: Integrative Analysis of the Metabolome and Transcriptome Provides Insights into the Mechanisms of Flavonoid Biosynthesis in Quinoa Seeds at Different Developmental Stages
Source: Metabolites. 2022 Sep 22;12(10):887. doi: 10.3390/metabo12100887 (PMC9609036; doi:10.3390/metabo12100887)
Supplement: Supplementary file 1 [file metabolites-12-00887-s001.zip › Figure.S6.pdf]

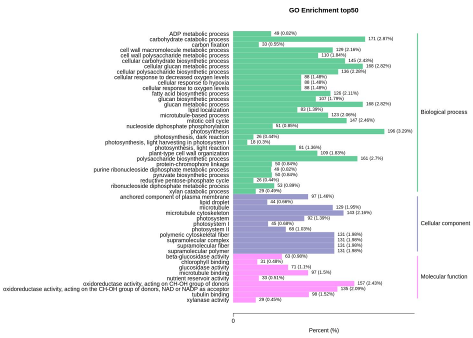

FB2 vs. RB2

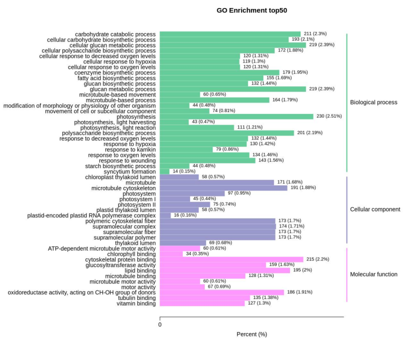

FB2 vs. DB2

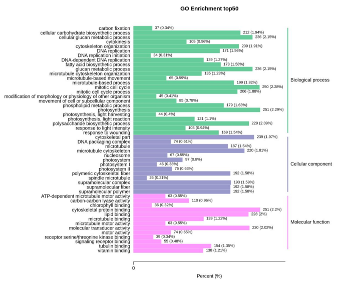

FB2 vs. MB2

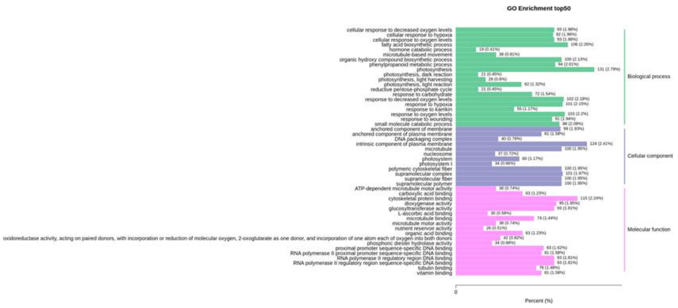

RB2 vs. DB2

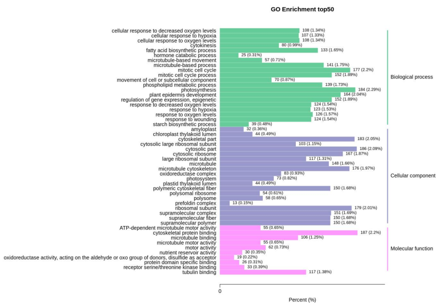

RB2 vs. MB2

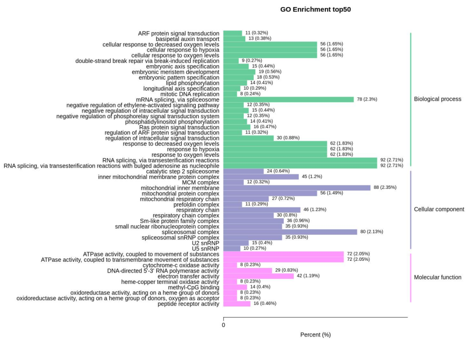

DB2 vs. MB2

Figure S6. Column diagram of differential gene GO enrichment The abscissa represents the proportion of genes annotated in a given entry to the total number of genes annotated, and the ordinate represents the name of the GO entry. The label on the right side of the figure represents the classification to which the GO item belongs.
