# Supplementary material for: Integrative Analysis of the Metabolome and Transcriptome Provides Insights into the Mechanisms of Flavonoid Biosynthesis in Quinoa Seeds at Different Developmental Stages
Source: Metabolites. 2022 Sep 22;12(10):887. doi: 10.3390/metabo12100887 (PMC9609036; doi:10.3390/metabo12100887)
Supplement: Supplementary file 1 [file metabolites-12-00887-s001.zip › Figure.S7.pdf]

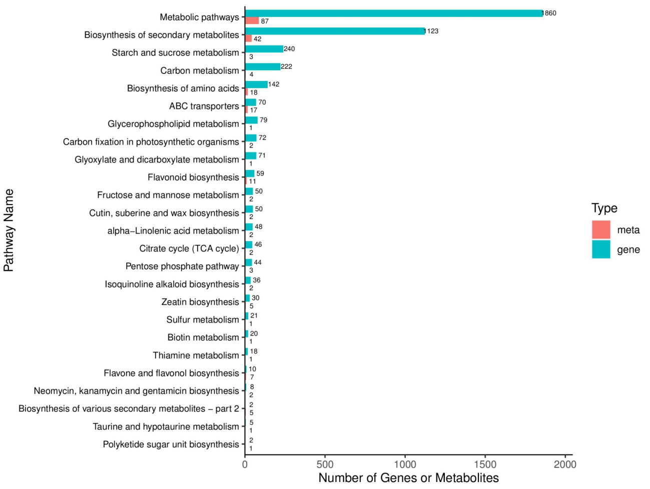

FB2 VS. RB2

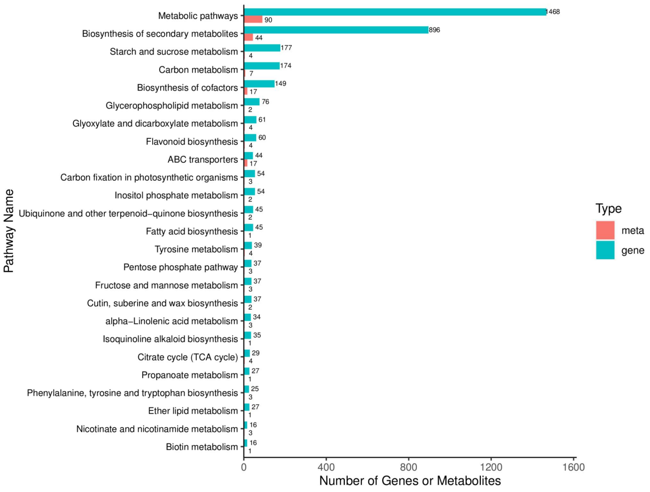

RB2 VS. DB2

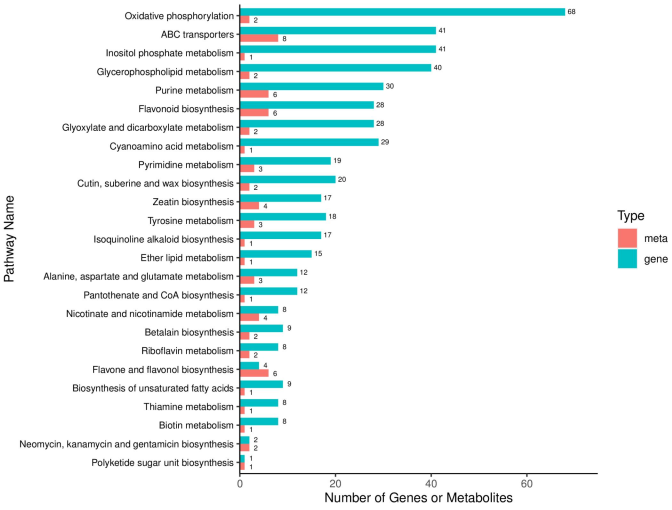

DB2 VS. MB2

Figure S7. KEGG enrichment analysis bar chart. Note: the abscissa represents the number of differential metabolites and differential genes enriched in this pathway, the ordinate represents the name of KEGG pathway, and the red and green bars represent metabolome and transcriptome respectively
