# Supplementary material for: Integrative Analysis of the Metabolome and Transcriptome Provides Insights into the Mechanisms of Flavonoid Biosynthesis in Quinoa Seeds at Different Developmental Stages
Source: Metabolites. 2022 Sep 22;12(10):887. doi: 10.3390/metabo12100887 (PMC9609036; doi:10.3390/metabo12100887)
Supplement: Supplementary file 1 [file metabolites-12-00887-s001.zip › Figure.S8.pdf]

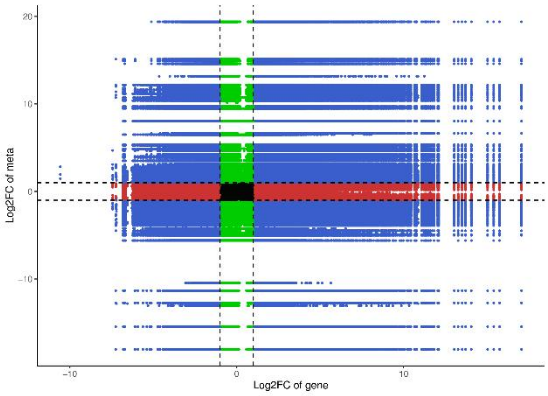

FB2 VS. RB2

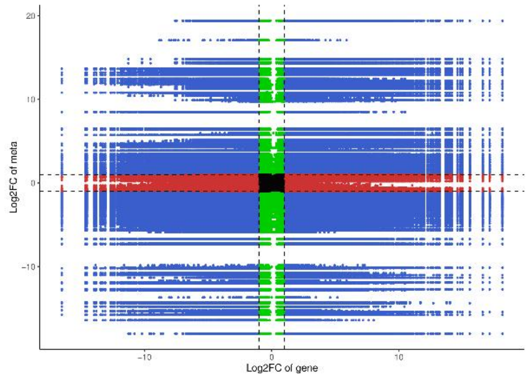

FB2 VS. DB2

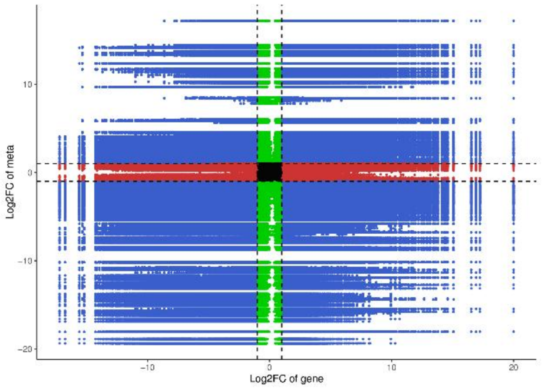

FB2 VS. MB2

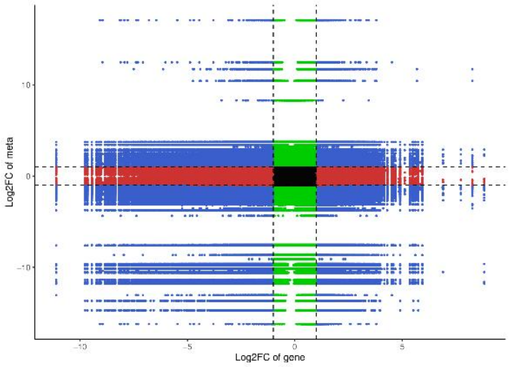

RB2 VS. DB2

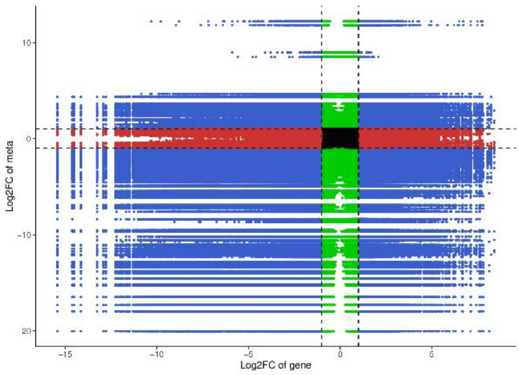

RB2 VS. MB2

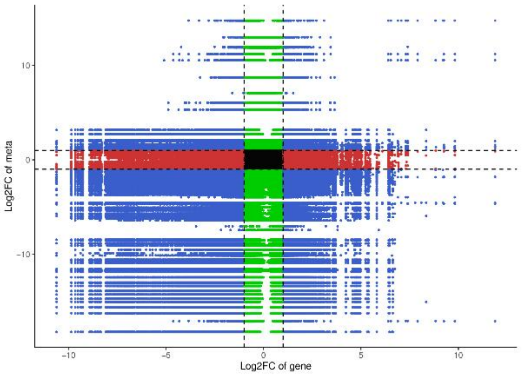

DB2 VS. MB2

Figure S8. Correlation analysis and nine-quadrant chart. The black dotted line is divided into quadrants 1–9 from left to right and from top to bottom. For quadrant 5, the genes, metabolites, and differently grouped genes and metabolites are not differentially expressed. For quadrants 3 and 7, the genes and metabolites have the same differential expression patterns. For genes and metabolites with consistent expression level trends, the genes may positively regulate the changes in the metabolites. For quadrants 1 and 9, the genes and metabolites have the opposite differential expression patterns. For genes and metabolites with inconsistent expression level trends, the genes may negatively regulate the changes in the metabolites. For quadrants 2, 4, 6, 8, when the metabolites are unchanged, the genes are upregulated or downregulated and when the genes are unchanged, the metabolites are upregulated or downregulated.
