# Supplementary material for: Integrative Analysis of the Metabolome and Transcriptome Provides Insights into the Mechanisms of Flavonoid Biosynthesis in Quinoa Seeds at Different Developmental Stages
Source: Metabolites. 2022 Sep 22;12(10):887. doi: 10.3390/metabo12100887 (PMC9609036; doi:10.3390/metabo12100887)
Supplement: Supplementary file 1 [file metabolites-12-00887-s001.zip › Figure.S9.pdf]

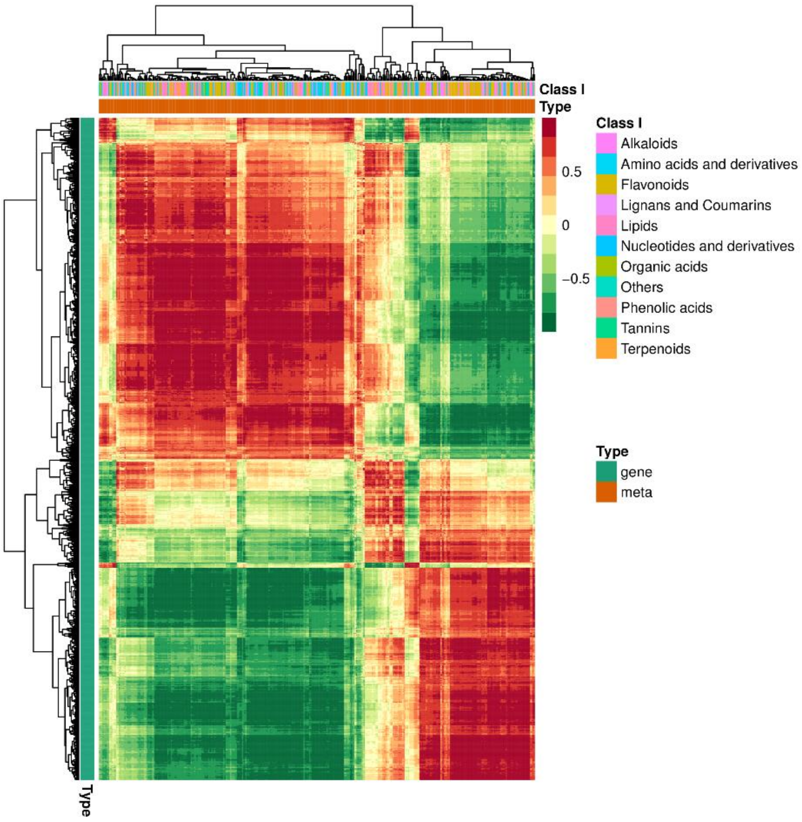

FB2 VS. RB2

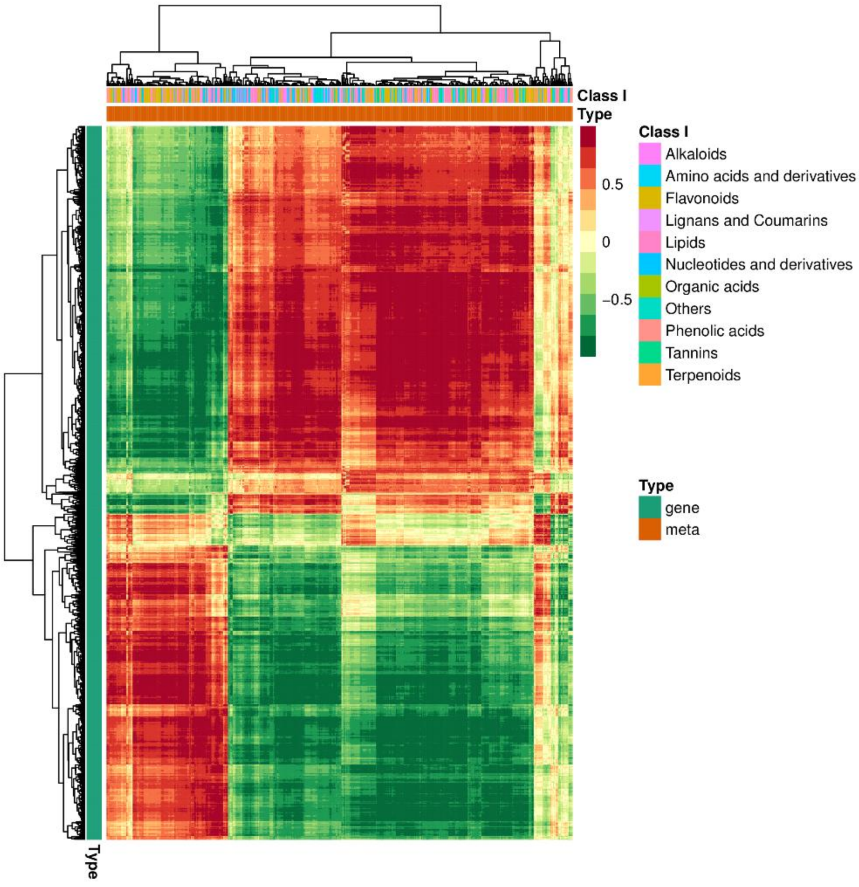

FB2 VS. DB2

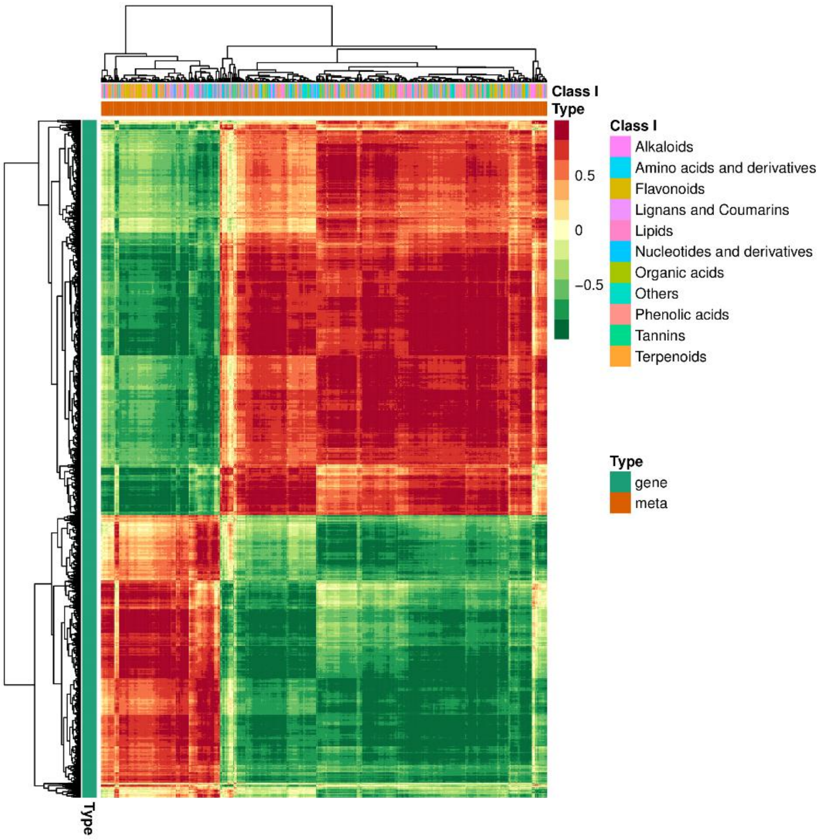

FB2 VS. MB2

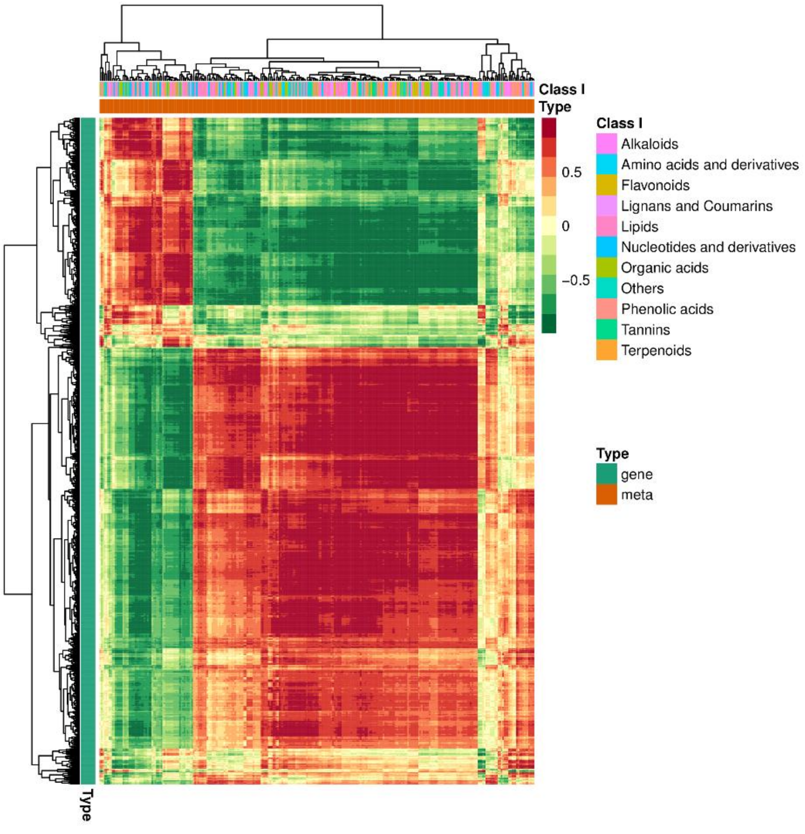

RB2 VS. DB2

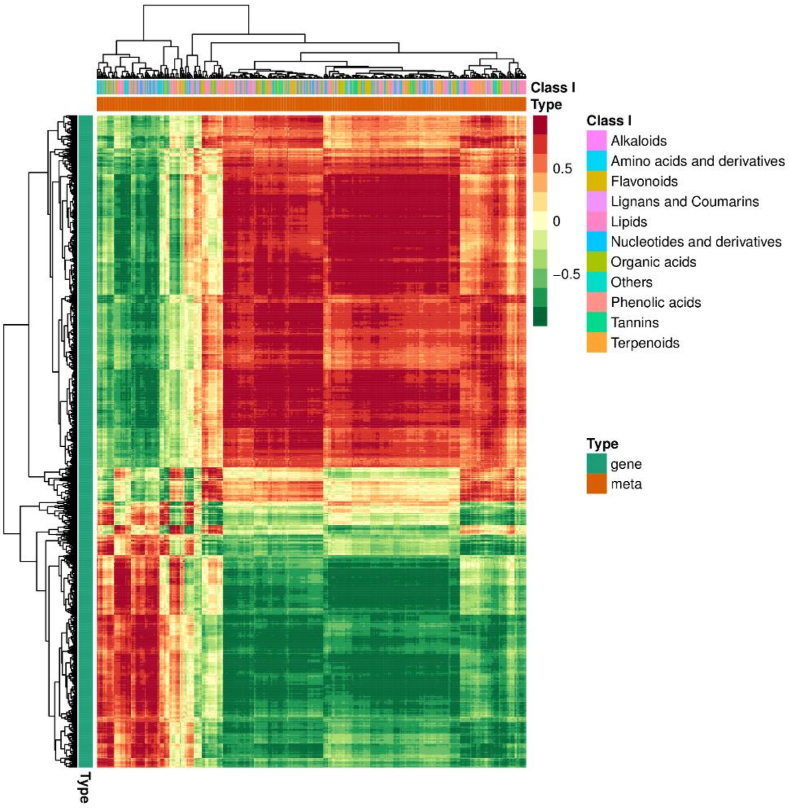

RB2 VS. MB2

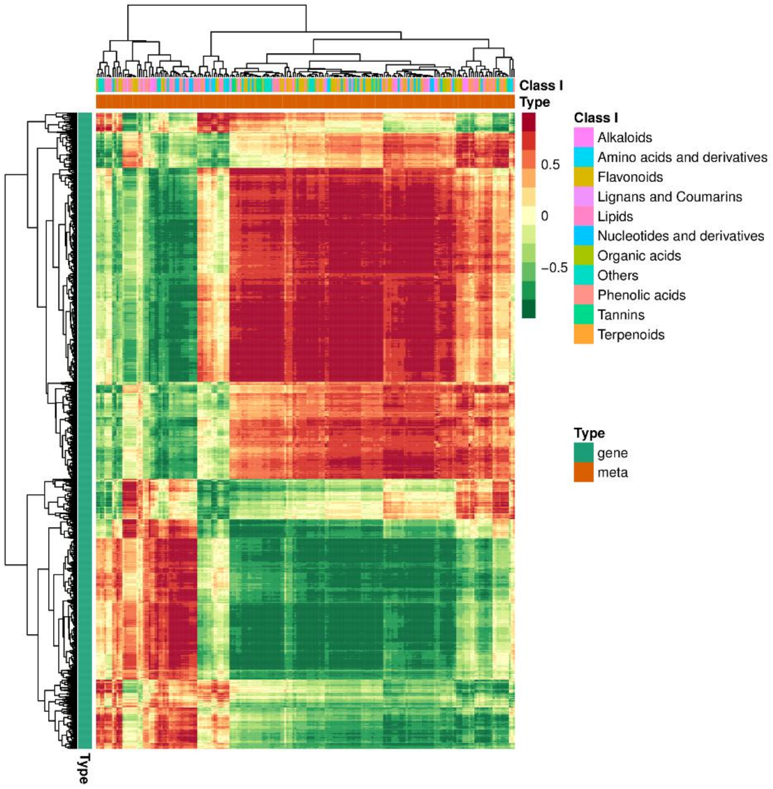

DB2 VS. MB2

Figure S9. Correlation Clustering heat map. Note: each row in the figure is a gene, and each column is a metabolite. Red represents a positive correlation between genes and metabolites, and green represents a negative correlation between genes and metabolites
