# Supplementary figures and images for: Integrative Analysis of the Metabolome and Transcriptome Provides Insights into the Mechanisms of Flavonoid Biosynthesis in Quinoa Seeds at Different Developmental Stages
Source: Metabolites. 2022 Sep 22;12(10):887. doi: 10.3390/metabo12100887 (PMC9609036; doi:10.3390/metabo12100887)

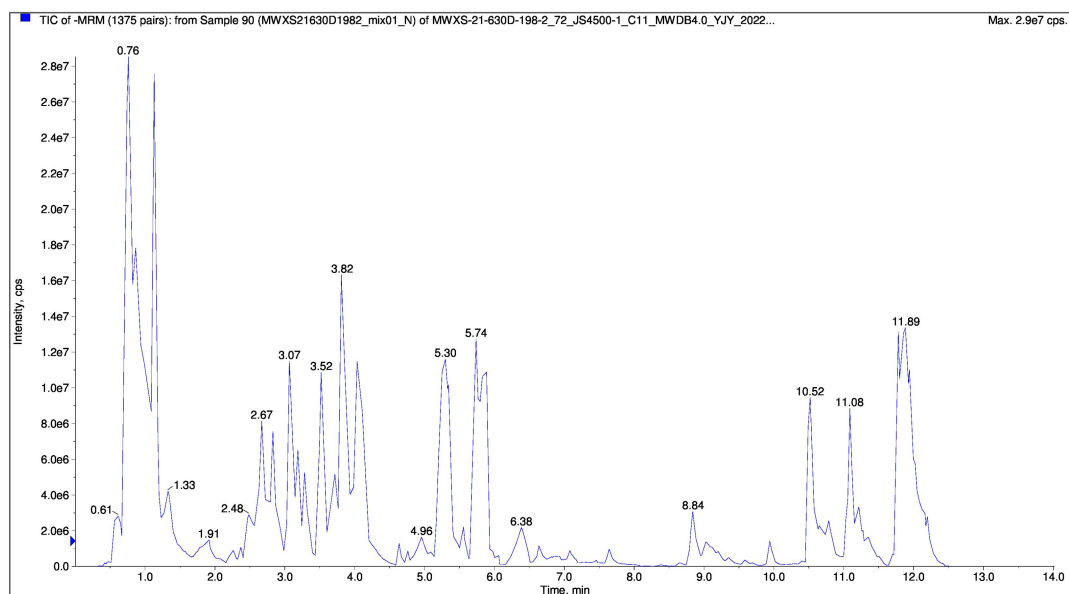

N

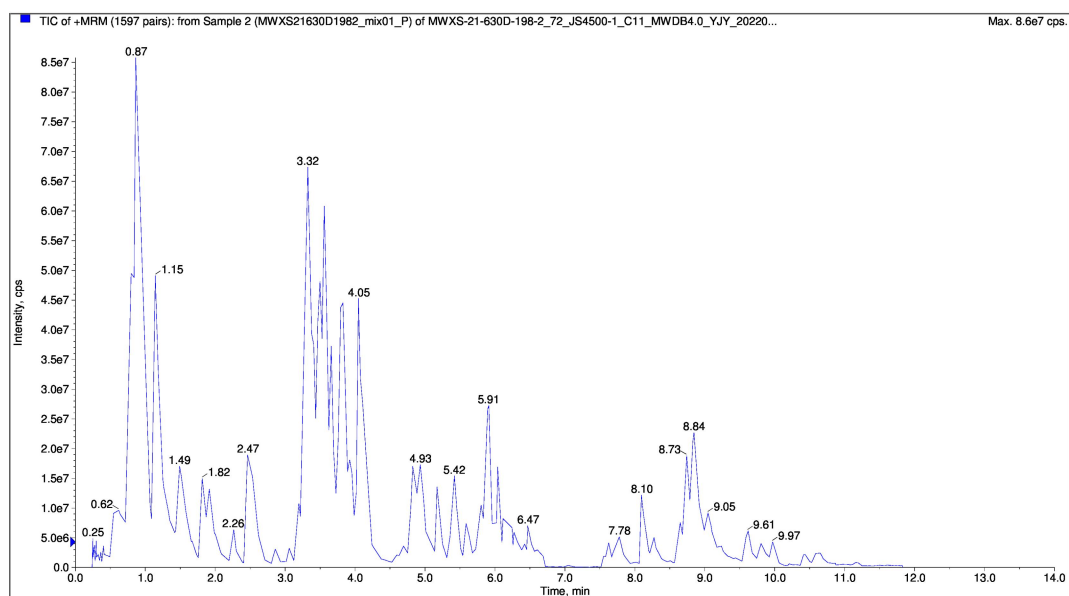

P

Figure S2. Total ion flow (TIC) showing positive and negative ion modes (from (top) to (bottom)).

Supplement: Supplementary file 1 [file metabolites-12-00887-s001.zip › Figure.S2.pdf]
